# Inhibitory effects of *Acanthopanax sessiliflorus* Harms extract on the etiology of rheumatoid arthritis in a collagen-induced arthritis mouse model

**DOI:** 10.1186/s13075-023-03241-1

**Published:** 2024-01-02

**Authors:** Dahye Kim, Yunji Heo, Mangeun Kim, Godagama Gamaarachchige Dinesh Suminda, Umar Manzoor, Yunhui Min, Minhye Kim, Jiwon Yang, Youngjun Park, Yaping Zhao, Mrinmoy Ghosh, Young-Ok Son

**Affiliations:** 1 Division of Animal Genetics and Bioinformatics, National Institute of Animal Science, RDA, Wanju, Republic of Korea; 2 Department of Animal Biotechnology, Faculty of Biotechnology, College of Applied Life Sciences, Jeju National University, Jeju City, Jeju Special Self-Governing Province 63243 Republic of Korea; 3 Interdisciplinary Graduate Program in Advanced Convergence Technology and Science, Jeju National University, Jeju City, Jeju Special Self-Governing Province 63243 Republic of Korea; 4 Laboratory of Immune and Inflammatory Disease, College of Pharmacy, Jeju Research Institute of Pharmaceutical Sciences, Jeju National University, Jeju, 63243 Republic of Korea; 5 Frontiers Science Center for Transformative Molecules, School of Chemistry and Chemical Engineering, Shanghai Jiao Tong University, Shanghai, 200240 People’s Republic of China; 6 Department of Biotechnology, School of Bio, Chemical and Processing Engineering (SBCE), Kalasalingam Academy of Research and Education, Krishnankoil, Srivilliputhur 626126 India; 7 Practical Translational Research Center, Jeju National University, Jeju, 63243 Republic of Korea

**Keywords:** Acanthopanax sessiliflorus harms, Rheumatoid arthritis, Supercritical carbon dioxide, Gene ontology, Protein–protein interaction network, Collagen-induced rheumatoid arthritis

## Abstract

**Background:**

The biological function of Acanthopanax sessiliflorus Harm (ASH) has been investigated on various diseases; however, the effects of ASH on arthritis have not been investigated so far. This study investigates the effects of ASH on rheumatoid arthritis (RA).

**Methods:**

Supercritical carbon dioxide (CO_2_) was used for ASH extract preparation, and its primary components, pimaric and kaurenoic acids, were identified using gas chromatography-mass spectrometer (GC–MS). Collagenase-induced arthritis (CIA) was used as the RA model, and primary cultures of articular chondrocytes were used to examine the inhibitory effects of ASH extract on arthritis in three synovial joints: ankle, sole, and knee.

**Results:**

Pimaric and kaurenoic acids attenuated pro-inflammatory cytokine-mediated increase in the catabolic factors and retrieved pro-inflammatory cytokine-mediated decrease in related anabolic factors in vitro; however, they did not affect pro-inflammatory cytokine (IL-1β, TNF-α, and IL-6)-mediated cytotoxicity. ASH effectively inhibited cartilage degradation in the knee, ankle, and toe in the CIA model and decreased pannus development in the knee. Immunohistochemistry demonstrated that ASH mostly inhibited the IL-6-mediated matrix metalloproteinase. Gene Ontology and pathway studies bridge major gaps in the literature and provide insights into the pathophysiology and in-depth mechanisms of RA-like joint degeneration.

**Conclusions:**

To the best of our knowledge, this is the first study to conduct extensive research on the efficacy of ASH extract in inhibiting the pathogenesis of RA. However, additional animal models and clinical studies are required to validate this hypothesis.

**Supplementary Information:**

The online version contains supplementary material available at 10.1186/s13075-023-03241-1.

## Background

Arthritis is a prevalent health concern affecting millions of people globally and is a leading cause of disability [1, 2]. Arthritis is not limited to the elderly, and more than three in five patients diagnosed are under the age of 65. Arthritis includes a group of more than 100 distinct diseases that fall into two major categories: osteoarthritis and inflammatory (or “autoimmune”) arthritis which affects people of all ages, irrespective of sex. Rheumatoid arthritis (RA) belongs to the category of inflammatory arthritis. This chronic condition can reduce a person’s life expectancy by approximately 3 to 18 years. If left untreated, 80% of the RA patients become work disabled after 20 years [3]. RA-associated arterial inflammation in patients increases the incidence of complications, such as cardiovascular disease and osteoporosis, and mortality owing to its direct and indirect effects on other systemic symptoms [4].

Over the past 30 years, there have been considerable changes in the early care and long-term management of RA [5]. Several effective drugs are available to treat RA; however, the cost of direct medical care may vary depending on the type of treatment. According to a survey, total medical care for a patient with RA costs US $12,509, with RA-specific treatments accounting for 30% of the total expense. The total direct medical expenditure among patients administered biologic disease-modifying antirheumatic medications was US $36,053, with RA-specific treatment costs accounting for 56% of the total expense [6, 7]. Unfortunately, current antirheumatic drugs do not improve the long-term prognosis of RA, have limited effectiveness, and numerous adverse consequences [8]. Therefore, it may be challenging to balance the dose and toxicity in each patient. High doses of these drugs can cause gastrointestinal irritation, gastrointestinal ulcerations, hemorrhagic events, and nephrotoxicity induced by nonsteroidal anti-inflammatory drugs (NSAIDs) [9–11]. Nevertheless, the role of present-day therapists is to relieve pain and not disease remission or a state of low disease activity. To overcome the limitations of synthetic medications; safer, easily accessible, highly potent, and economical therapeutic agents are needed to treat RA [12]. Constant attempts have been made to investigate the efficacy of medicinal plants and their phytochemicals, which have been used in traditional medicine [13]. Compared with nonsteroidal anti-inflammatory medications, phytomedicines for the treatment of pain have a broad range of action. Phytomedicines target diverse inflammatory and chondrodestructive pathways [14].

*Acanthopanax* is a plant genus native to East and South Asia, possesses ginseng-like activities, and is used in traditional medicine [15–17]. Extensive studies have revealed that phytochemicals such as lignans, diterpenoids, triterpenoids, phenylpropanoids, polyacetylenes, and flavonoids reported in *A. sessiliflorus* play key roles in the treatment of rheumatoid arthritis, diabetes, bacterial infections, cancer, and hypertension [18–21]. Pimaric and kaurenoic acids from *Acanthopanax* are primary intermediates in the biogenesis of gibberellins and other phytohormones that control plant growth, development, and various fungal metabolites [17, 22]. It exhibits substantial anti-inflammatory, antihypertensive, and diuretic biological effects in vivo and antibacterial, smooth muscle relaxant, and cytotoxic properties in vitro [23, 24]. Previous studies demonstrated the anti-inflammatory properties of pimaric acid [25]. Pimaric acid suppresses the synthesis of matrix metalloproteinase (MMP)-9 mRNA through MAPK and its promoter activity in human aortic smooth muscle cells [26]. However, the effects of *A. sessiliflorus* and its major compounds on arthritis has not yet been investigated. This study aims at providing comprehensive details for investigating novel traditional herbal medicine (THM) components, i.e., pimaric and kaurenoic acids, from *A. sessiliflorus* Harm (ASH) for the treatment of RA. We evaluated whether ASH exhibits protective effects against cartilage degradation in collagenase-injected knee joints and examined the effects of pimaric and kaurenoic acids on pro-inflammatory cytokine-induced catabolic expression, both in vitro and in vivo. Further study was conducted functional annotation cluster analysis on genes with fold changes. We employed DAVID for this purpose and used the EASE tool for gene ontology (GO) representation. Pathway mapping was done using the Kyoto Encyclopedia of Genes and Genomes (KEGG) tool. For creating detailed graphical representations of biological processes and gene pathways, we used the Biograph tool and the ToppGene suite with the Bonferroni correction method. Additionally, we inferred gene regulatory networks using a path consistency algorithm based on conditional mutual information.

## Materials and methods

### Reagents

Dulbecco’s Modified Eagle’s Medium and fetal bovine serum were purchased from Thermo Fisher Scientific (Grand Island, NY, USA). Chemicals, liquid chromatography-mass spectrometry grade water, and Falcon Labware were purchased from Sigma Chemical Co. (St. Louis, MO, USA). Taq polymerase was purchased from HanLAB (Cheongju-si, Chungcheongbuk-do, South Korea).

### Preparation of ASH extract via supercritical CO_2_

**Fig. 1:**
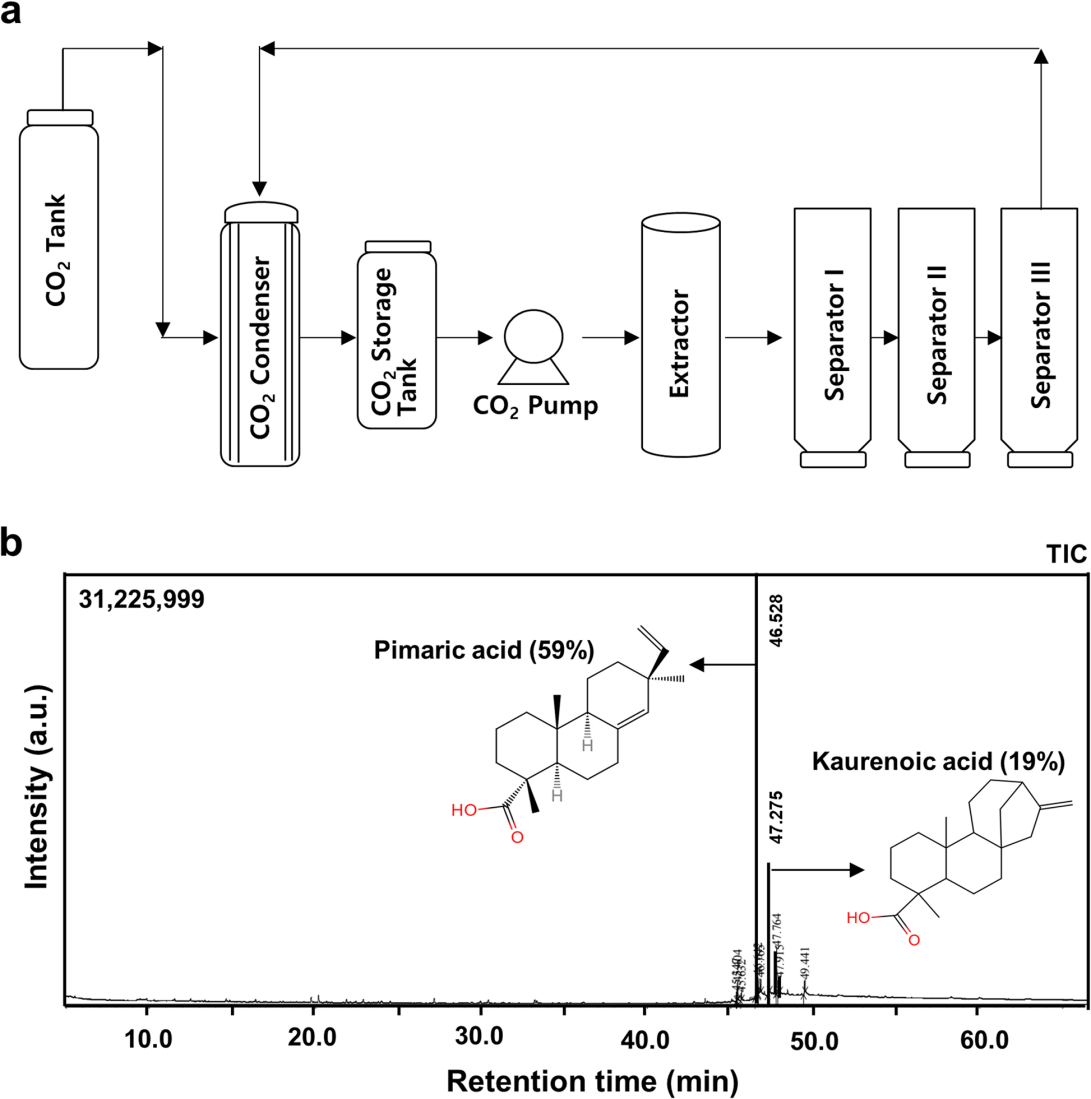
Schematic representation of the supercritical extraction apparatus and GC–MS chromatogram for A. sessiliflorus Harms extract using supercritical CO_2_

The supercritical CO_2_ extraction method was used for ASH extraction as previously mentioned [27]. The extraction apparatus was manufactured by Nantong Wisdom Supercritical Science and Technology Development Co., Ltd. (China) (Fig. 1). The system consists of a 5-L extractor, two separators (separator 1:3 L, separator 2:2 L), a chiller, and a high-pressure CO_2_ pump that supplies CO_2_ from the cylinder into the system. Heating jackets were used to heat the entire apparatus. A typical experiment involved loading 2 kg of powdered ASH into an extractor and pumping a specific amount of CO_2_ into the system. The system was subsequently emptied by opening valve 9 (V9). To replenish the air in the system, three CO_2_ purges were performed. All valves were shut off after purging, except for V1 and V2, such that a certain volume of CO_2_ could be injected into the extractor. V5, V8, and V11 were opened for CO_2_ circulation and dynamic extraction after the extractor pressure and temperature reached 40 MPa and 55 °C, respectively. Separators 1 and 2 were adjusted to 50 °C and 13 MPa and 40 °C and 5 MPa, respectively. The CO_2_ mass flow rate was maintained at 35 kg/h during the extraction, which lasted for 2 h. After extraction, the sample was collected using V7 and stored for subsequent testing.

### Analysis of ASH extract using gas chromatography-mass spectrometry (GC–MS)

The GC–MS analyses of ASH extracts were performed using a Shimadzu QP2010 SE gas chromatography-mass spectrometer (Shimadzu, Kyoto, Japan) equipped with a DB-1 capillary column (25 m in length × 0.32 mm in diameter × 0.52 µm in thickness) (Agilent, USA). Helium was used as the carrier gas at a constant flow rate of 0.5 mL/min. For GC–MS spectral detection, an electron ionization energy method was adopted with a high ionization energy of 70 eV. The injector temperature was set to 250 °C. The temperature of the column was set at 40 °C for 4 min and increased by 10 °C per minute up to 240 °C. Based on a comparison of the retention time (min), peak area, peak height, and mass spectrum of the phytochemicals present in the test samples and the mass spectral library database, we identified the phytochemical contents of the test samples.

### Experimental model of RA and von Frey and hot plate assays

All experiments were approved by the Institutional Animal Care and Committee of Jeju National University (2022–0002). To avoid any developmental defects resulting from hormonal differences, 7-week-old DBA/1 J male mice were used to develop experimental RA models. Under isoprene anesthesia, equal amounts of collagen type II and 4 mg/mL complete Freund’s adjuvant were combined and slowly injected into the skin of the tail. The von Frey assay was performed once a week for 7 days after arthritis induction. The filament stab began with a thick size on the sole and was recorded as the mouse lapped. Each stab was applied five times, and licking was performed thrice. The filament was replaced with a lower filament and processed for additional studies if the mouse did not respond [28]. The Plantar Test for Thermal Stimulation-Hargreaves Apparatus (ugo basile®, 37,570, Gemonio, Italy) was used to perform the hot plate test. The experimental mouse was positioned at five different spots, and the time taken to remove the mouse from the sole plate was recorded in seconds. For precise measurement, the response time was recorded when the mouse licked or popped. The average results were recorded five times for each leg [29, 30].

### IP (intraperitoneal) injection

ASH injections were started 1 week after collagenase-induced arthritis (CIA) induction. ASH (200 µg/µL) was diluted in polyethylene glycol 400 (PEG-400) and injected intraperitoneally twice a week at a concentration of 6 mg/kg per animal (200 µL). In the control group, 200 µL of PEG-400 was injected.

### Determination of the clinical score and paw thickness

After 1 week of arthritis induction by collagen, the severity and degree of swelling of the hind paws were checked every 3–4 days. Both paws were measured based on the hind paw of the mouse, and the clinical score was calculated using the Hooke Laboratories severity index. The clinical score scale was 0–4, and the symptoms were as follows: 0, normal; 1, swelling of 1 to 2 toes or no swelling of the feet; 2, swelling of 3 or more toes or slight swelling of the entire foot; 3, increased edema of the genitals; and 4, severe swelling of the developing body and inability to hold the cage with toes. To determine the paw thickness, both hind paws of mice were measured. The ankle was the measurement site, and measurements were taken only if swelling was observed. The measurements were performed using Vernier calipers.

### Histological examinations and calculation of the osteoarthritis research society international (OARSI) score

The tissues were fixed in 4% paraformaldehyde for 24 h before being decalcified in 0.5-M ethylenediaminetetraacetic acid for 2 weeks (pH 7.4). Following dehydration, the tissues were sectioned to a thickness of 4 μm for slide preparation. A tissue was embedded within a paraffin block [31]. Histological examinations were performed using Harris hematoxylin, fast green, and safranin O (all from Sigma-Aldrich, St. Louis, MO, USA) [32]. The histological degree of inflammation in RA was divided into two categories: OARSI grade (0–6) and pannus score (0–3) [11, 33–35]. Five observers assessed OARSI and pannus scores. The scoring results were derived by averaging the scores of the observers for each mouse. A typical image of safranin O with the most representative etiology among the parts was selected.

### Immune cell infiltration analysis

The infiltration of eosinophils and mast cells into the synovial membrane surrounding the joints was also investigated. Eosinophils and mast cells were stained with Congo red (Sigma C6277, St. Louis, MO, USA) and toluidine blue O (Sigma-Aldrich), respectively, and CD15 was applied for neutrophil extracellular traps. ImageJ software (NIH) was used to quantify immune cells.

### Reverse transcription-polymerase chain reaction (RT-PCR) for chondrocyte RNA

Chondrocytes were extracted from the femoral condyles and tibial plateaus of mice 5 days post birth employing 0.2% collagenase digestion method [36–38]. The TRIzol reagent was used to extract total RNA from primary cultured chondrocytes (Molecular Research Center Inc., Cincinnati, OH, USA). The NanoDrop™ 2000 Spectrophotometer (Thermo Scientific, Waltham, MA, USA) was used to assess the purity and concentration of RNA. The RNA was reverse-transcribed, and the resulting cDNA was amplified via PCR using the CFX96™ Real-Time System (Bio-Rad Laboratories, Inc., Hercules, CA, USA, at the Bio-Health Materials Core-Facility, Jeju National University). Target bands were quantified using the ImageJ densitometry software (NIH, Bethesda, MD, USA). GAPDH was used as the internal control. The PCR primers and experimental conditions are summarized in Table 1.

**Table 1:** List of genes and the primer details

Genes	Strand	Primer sequences	Size (bp)	A_T_ (°C)	Origin
Gapdh	A AS	5′-TCACTGCCACCCAGAAGAC-3′ 5′-TGTAGGCCATGAGGTCCAC-3′	450	57.3	Mouse
Mmp3	A AS	5′-AGGGATGATGATGCTGGTATGG-3′ 5′-CCATGTTCTCCAACTGCAAAGG-3′	434	58.0	Mouse
Mmp10	A AS	5′-AGAAATGGACACTTGCACCCTCAG-3′ 5′-CTGTCCGTGTTGTGAGCCTCATAG-3′	448	61.5	Mouse
Mmp13	A AS	5′-TGATGGACCTTCTGGTCTTCTGG-3′ 5′-CATCCACATGGTTGGGAAGTTCT-3′	474	58.2	Mouse
Adamts4	A AS	5′-CATCCGAAACCCTGTCAACTT-3′ 5′-GCCCATCATCTTCCACAATAGC-3′	287	58.4	Mouse
Adamts5	A AS	5′-GCCATTGTAATAACCCTGCACC-3′ 5′-TCAGTCCCATCCGTAACCTTTG-3′	292	58.4	Mouse
Aggrecan	A AS	5′-GAAGACGACATCACCATCCAG-3′ 5′-CTGTCTTTGTCACCCACACAT-3′	581	60.0	Mouse
Col2a1	A AS	5′-CACACTGGTAAGTGGGGCAAGACCG-3′ 5′-GGATTGTGTTGTTTCAGGGTTCGGG-3′	173	58.0	Mouse
Sox9	A AS	5′-ATGCTATCTTCAAGGCGCTG-3′ 5′-GACGTCGAAGGTCTCAATGT-3′	272	60.0	Mouse

### Western blotting

The protein content of each sample was quantified via BCA analysis, and 50 µg of protein per sample was separated using 10–12% Bis–Tris gel (Bio-Rad Laboratories, Inc.). The isolated protein was subsequently transferred to the Odyssey® Nitrocellulose Membrane (LI-COR, Lincoln, NE, USA) and blocked with Intercept® (TBS) Blocking Buffer (LI-COR), followed by Intercept™ (TBS) Antibody Diluent T20 (primary antibody to LI-COR): COX-2 (Santa Cruz Biotechnology Inc., Santa Cruz, CA, USA); β-actin (Solarbio, Beijing, China); p65, p-p65, IkBa, and p-IkBa (Cell Signaling Technology, Inc., Danvers, MA, USA); and dilute iNOS (Abcam, Cambridge, UK), and incubated at 4 °C for 12 ~ 16 h. The secondary antibody (LI-COR) was diluted in Intercept™ (TBS) Antibody Diluent T20 (LI-COR), blocked in the dark, and probed at 20 °C for 1 h. The membrane was completely dried in dark, and the bands were detected using the Odyssey® DLx Imaging System (LI-COR).

### Annotation of pathway and information-based network inference of genes

Functional annotation cluster analysis was conducted using DAVID (www.david.ncifcrf.gov), considering the list of genes with fold changes. The expression analysis systematic explorer (EASE) tool from DAVID was used to investigate Gene Ontology (GO) representations of functional groups. The Kyoto Encyclopedia of Genes and Genomes (KEGG) tool was used to map the pathways. The Biograph tool (http://www.biograph.be) and ToppGene suite (https://www.toppgene.cchmc.org/; Bonferroni correction method) were used to create detailed graphical representations of biological processes and gene pathway analysis in datasets involving multiple sets of genes and to perform systems biology-based dissection of biological states. We also inferred gene regulatory networks from gene expression data using a path consistency algorithm based on conditional mutual information (PCACMI) [39, 40] and enhanced the construction of gene regulatory networks using hub gene information conditional mutual inclusive information-based network inference (CMI2NI) [40, 41].

More precisely, A(*X*) $[{\text{i}}.{\text{e}}., A\left(X\right)=E\left(-{\text{log}}fx\left(X\right)\right)]$ and A (*X, Y*)$[{\text{i}}.{\text{e}}., A\left(X, Y\right)=E\left(-{\text{log}}{f}_{xy}\left(XY\right)\right)]$ represent the entropies of random variables *X* and *Y* and their combined entropies. While MI cannot differentiate between direct and indirect reliance, conditional mutual information (CMI) can measure the direct dependency between two variables by conditioning other variables, i.e., Z. A. Gaussian kernel density estimator was used to calculate entropies. The MI and CMI are defined as follows:

1 $$\widehat{I}\left(X,Y\right)=\frac{1}{2}{\text{log}}\frac{\left|C(X)\right|\left|C(Y)\right|}{\left|C(X,Y)\right|}$$

2 $$\widehat{I}\left(X,Y\left|Z\right|\right)=\frac{1}{2}{\text{log}}\frac{\left|C(X,Z)\right|\left|C(Y,Z)\right|}{\left|C(Z)\right|\left|C(X,Y,Z)\right|}$$

C(X), C(Y), and C(Z) are the variances of X, Y, and Z, respectively, and C(X, Z), C(Y, Z), and C(X, Y, Z) are the covariance matrices of (X, Z), (Y, Z), and (X, Y, Z), respectively.

Furthermore, instead of using CMI, the CMI2NI method uses conditional mutual inclusive information (CMI2) as a measure of the reliance between two variables of interest in the context of additional variables. Considering three random variables, *X*, *Y*, and *Z*, the CMI2 between *X* and *Y* at given *Z* is defined as CMI2(X, Y|Z|) = (D_KL_(P||P _X->Y_ + D_KL_ (P||P _Y->X_))/2, where (D_KL_(*f*||g) is the Kullback–Leibler divergence from *f* and *g*, P is considered the join PDF of *X*, *Y*, *Z*, and* P*_Y->X_ is the interventional probability of *X*, *Y*, and *Z* for removing the connection from *X* to *Y*. CMI2 for two random variables *X* and *Y*, given the m-dimensional vector Z, can be defined using the Gaussian hypothesis:

3 $$CMI2(X,Y|Z|) =\frac{1}{4} (tr \left({C}^{-1}\sum )+tr({\overline{C} }^{-1} \overline{\Sigma }\right)+{logC}_{0}+ {log\overline{C} }_{0}- 2n)$$

where ∑ and $\overline{\Sigma }$ are the covariance matrix of (*X*, *Y*, *Z*^T^)^T^.

### Statistical analyses

All statistical analyses were performed using IBM SPSS Statistical software (IBM Corp., Armonk, NY, USA). To compare data from the cell-based in vitro experiments for pairwise comparisons and multiple comparisons, two-tailed Student’s *t*-tests with uneven sample sizes and variances and two-way ANOVA with post hoc testing (LSD) were used, respectively. The OARSI grade from the histological experiments was analyzed using the nonparametric Mann–Whitney *U*-test. The Shapiro–Wilk test was used to verify that the distribution was normal. The “n” indicates the number of separate experiments or animals. The cutoff for statistical significance was *p* < 0.05.

## Results

### Identification of phytochemicals extracted from *A. sessiliflorus* Harms

The GC–MS chromatogram of ASH methanol extract showed 10 peaks corresponding to the bioactive compounds (Fig. 1b and Table 2), exhibiting various phytochemical activities that were identified by presenting their peak retention time, peak area (%), height (%), and mass spectral fragmentation patterns comparable to the online library search. According to the peak area (%) and reference history, 2 major compounds out of 10 were identified (pimaric and kaurenoic acids).

**Table 2:** List of compounds in *A sessiliflorus* harms extract

No.	Compound (IUPAC)	Chemical formula	Retention time	Area %	Synonyms
1	Ethyl octadeca-9,12-dienoate	C_20_H_36_O_2_	45.347	1.10	Ethyl linoleate
2	[(1S,4S,9S,10R,13S)-5,9-Dimethyl-14-methylidene-5-tetracyclo [11.2.1.01,10.04,9]hexadecanyl]methanol	C_20_H_32_O	45.404	2.16	(-)-kaur-16-en-19-ol
3	Ethyl octadecanoate	C_20_H_40_O_2_	45.632	0.82	Ethyl stearate
4	(1R,4aR,4bS,7S,10aR)-7-Ethenyl-1,4a,7-trimethyl-3,4,4b,5,6,9,10,10a-octahydro-2H-phenanthrene-1-carboxylic acid	C_20_H_30_O_2_	46.528	58.77	Pimaric acid
5	Methyl (1R,4aR,4bS,7S,10aR)-7-ethenyl-1,4a,7-trimethyl-3,4,4b,5,6,8,10,10a-octahydro-2H-phenanthrene-1-carboxylate	C_21_H_32_O_2_	46.642	2.95	Methyl isopimarate
6	(Z)-Octadec-9-enamide	C_18_H_35_NO	46.765	2.56	9-Octadecenamide
7	(1S,4S,5R,9S,10R,13S)-5,9-Dimethyl-14-methylidenetetracyclo[11.2.1.01,10.04,9]hexadecane-5-carboxylic acid	C_20_H_30_O_2_	47.275	18.63	Kaurenoic acid
8	1,3-Dihydroxypropan-2-yl hexadecanoate	C_19_H_38_O_4_	47.764	8.49	Palmitin, 2-mono
9	2-(2-Ethylhexoxycarbonyl)benzoic acid	C_16_H_22_O_4_	47.915	2.08	Phthalic acid mono-2-ethylhexyl ester
10	1,3-Dihydroxypropan-2-yl octadecanoate	C_21_H_42_O_4_	49.441	2.44	2-Stearoylglycerol

### The inhibitory effects of ASH in a CIA model representing RA

**Fig. 2:**
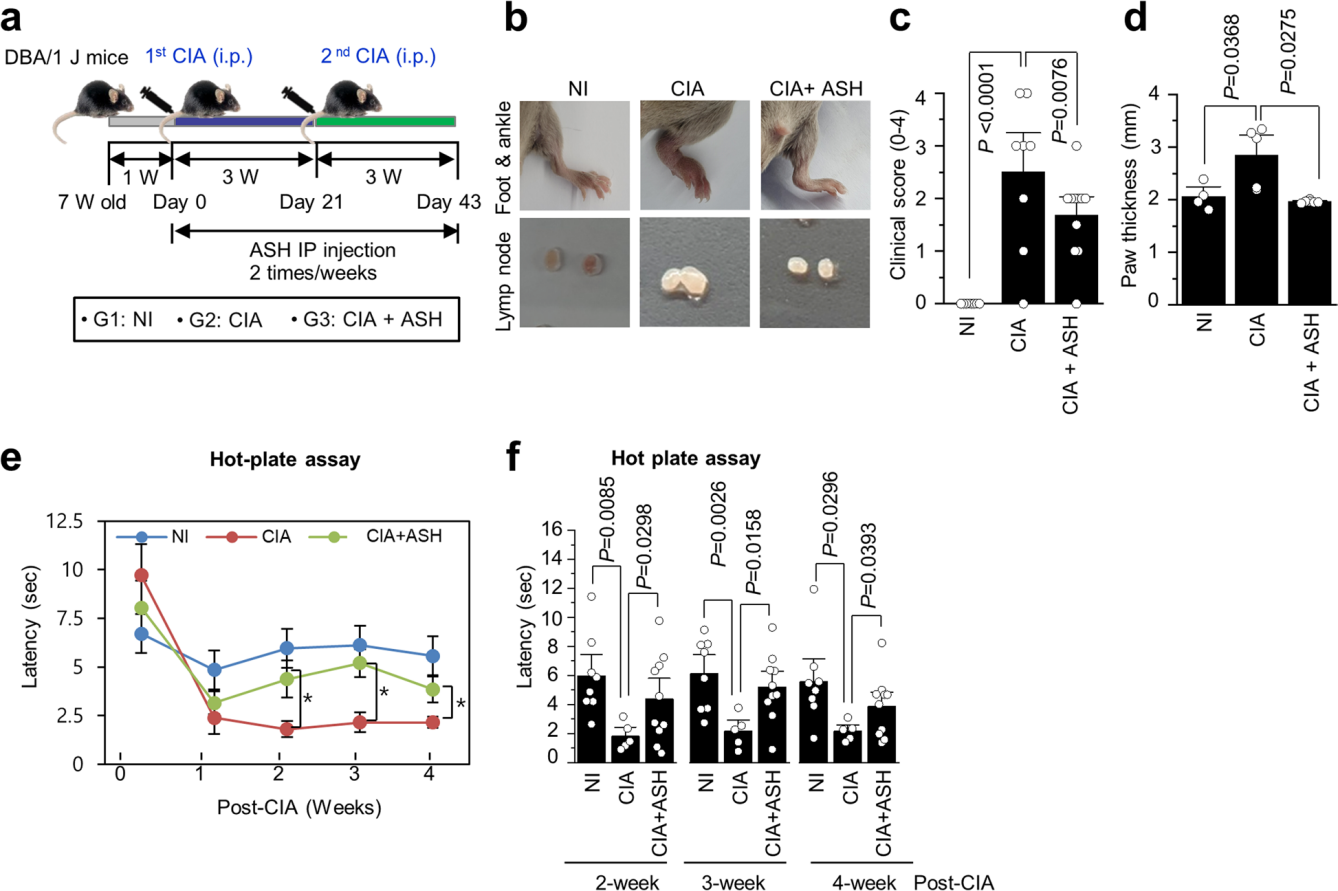
The inhibitory effects of A. sessiliflorus Harms extract on clinical symptoms and pain. (**a**) Outline of the experimental plan and animal grouping. (**b**) Differences in paw swelling among the experimental groups. Comparative study of (**c**) clinical scores and (**d**) paw thickness among the experimental groups. (**e** and **f**) Hot plate assay in a collagen-induced arthritis (CIA) model at 2, 3, and 4 weeks post arthritis induction

The ASH-treated group showed considerably lower severity than those treated with PEG alone (vehicle). The experimental setup is illustrated in Fig. 2a. The clinical RA symptoms were considerably lower in the ASH-treated group than that in the vehicle group (Fig. 2b). ASH administration also decreased the CIA-induced clinical score (*p* = 0.0076) and paw edema (*p* = 0.0275) (Fig. 2c and d). After immunization with collagen, the hot plate assay showed that, compared to the CIA group, the ASH group showed significantly reduced latency (Fig. 2e and f). The analgesic effect was significant at 2 weeks (*p* = 0.0298), 3 weeks (*p* = 0.0158), and 4 weeks (*p* = 0.0393) after administration.

### The effects of ASH extract on cartilage degeneration and pannus formation

**Fig. 3:**
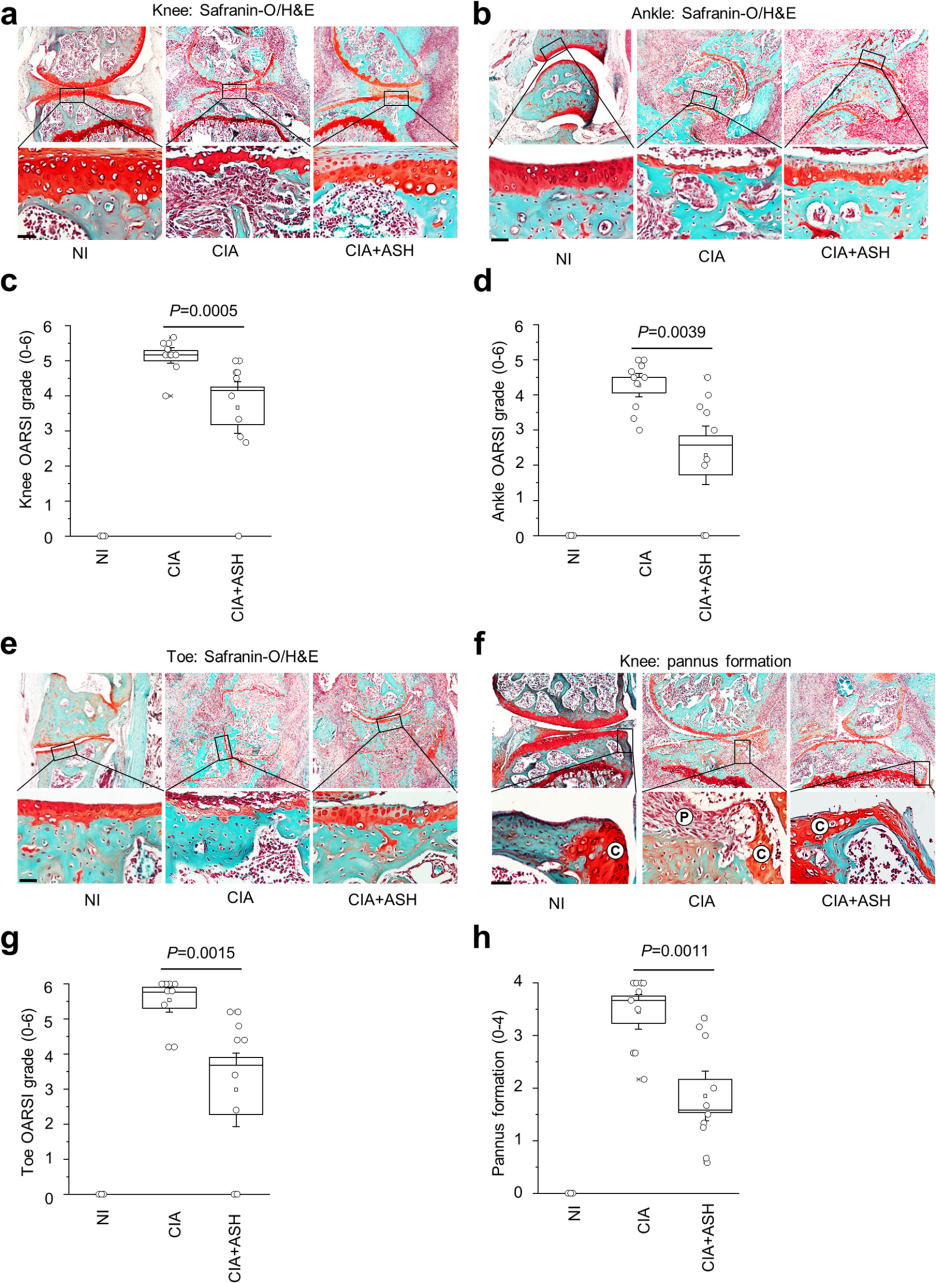
*A. sessiliflorus* Harms extract attenuates cartilage destruction and pannus formation in CIA model. Cartilage damage was visualized by safranin-O staining in (**a**) knees, (**b**) ankle, and (**e**) toe. The OARSI results show that the phyto-extract significantly reduces knee cartilage breakdown and erosion in the (**c**) knees, (**d**) ankle, and (**g**) toe of the treated groups. Safranin-O staining revealed that ASH significantly attenuates pannus formation in mice knees in CIA model (**f**, **h**)

The ASH extract decreased CIA-induced cartilage degradation and pannus development. The degree of cartilage deterioration and inflammation-mediated cell penetration into the bone was detected using safranin-O staining (Fig. 3a, b, e, f). Based on the ankle OARSI grade, ASH administration significantly decreased cartilage deterioration in the knee (*p* = 0.0005, Fig. 3c), ankle (*p* = 0.0039, Fig. 3d), and toe (*p* = 0.0015, Fig. 3g). However, the ASH extract nonsignificantly attenuated synovial membrane inflammation in the knee, ankle, and toe. Furthermore, safranin-O staining of the knees demonstrated that pannus formation was significantly reduced in the knees of ASH-treated mice (Fig. 3f, h; *p* = 0.0011) compared to PEG-treated mice, while nonsignificant effects were observed in mouse toes.

### The inhibitory effects of immune cells infiltrations by ASH in the CIA model representing RA

**Fig. 4:**
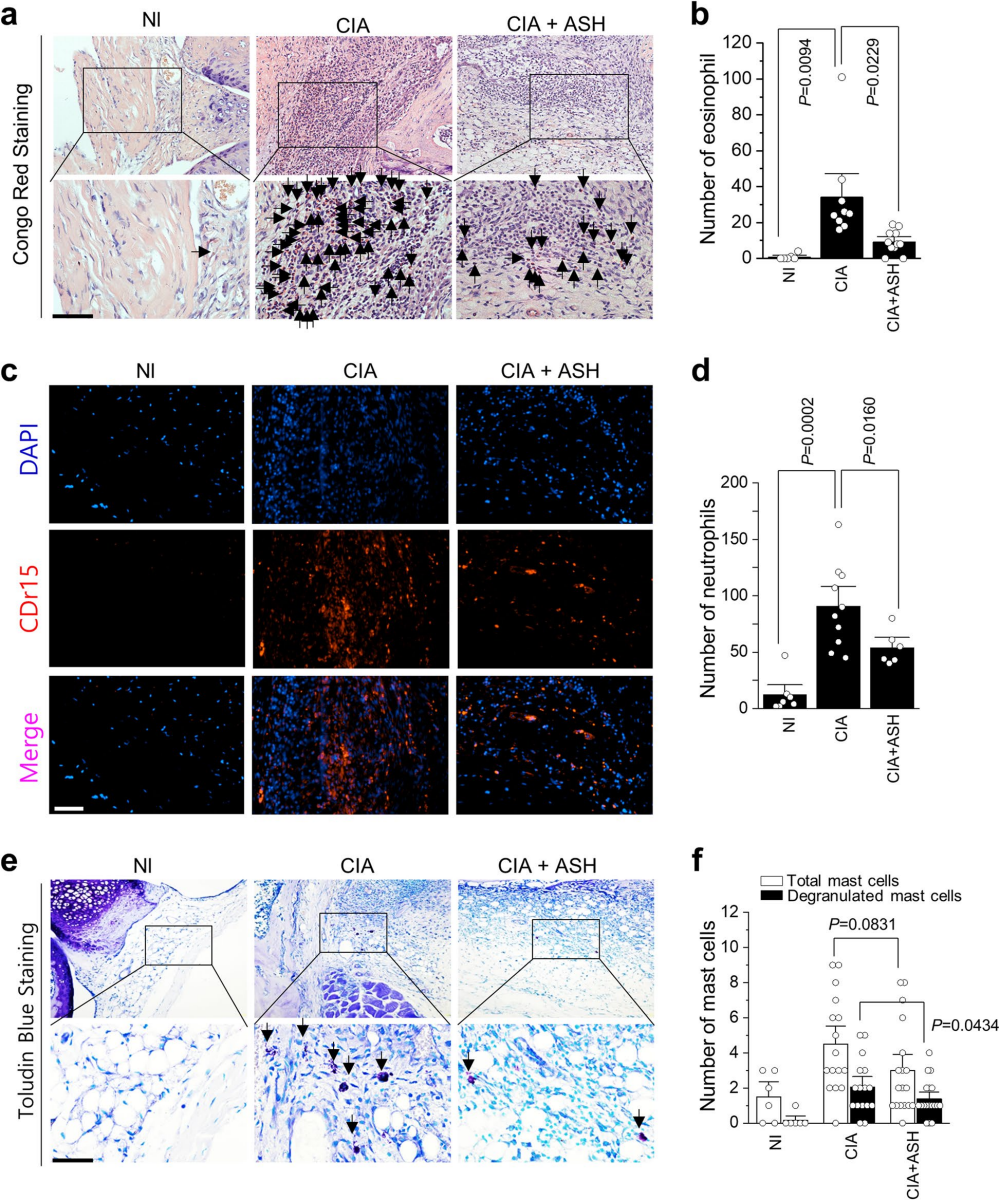
*A. sessiliflorus* Harms extract attenuates immune cell activation in a CIA model. (**a**) The eosinophil infiltration was analyzed using Congo red staining, (**b**) the quantification of eosinophil infiltration, (**c**) neutrophil penetration of the knee via CDr15 staining, and (**d**) the quantification of neutrophil infiltration; (**e**) mast cell number and activity in the knee were analyzed via toluidine blue staining; (**f**) total or degranulated mast cells were counted

Congo red staining indicated eosinophil infiltration into knee synovial membranes in the CIA group (Fig. 4a). Eosinophil counts were significantly reduced in comparison with those in the untreated PEG groups (*p* = 0.0229) (Fig. 4b). CDr15 immunostaining demonstrated that neutrophils had infiltrated the experimental groups (Fig. 4c). Neutrophil counts were significantly attenuated in the knees of the treated group (*p* = 0.016) (Fig. 4d). Toluidine blue staining revealed the presence of mast cells (Fig. 4e). The number of mast cells was reduced in the ASH-treated group compared to that in the CIA group (*p* = 0.0831), whereas degranulated mast cells were significantly attenuated (*p* = 0.0434) in the ASH-treated group compared to that in the untreated CIA group (Fig. 4f).

### The effects of ASH extract on pro-inflammatory cytokines

**Fig. 5:**
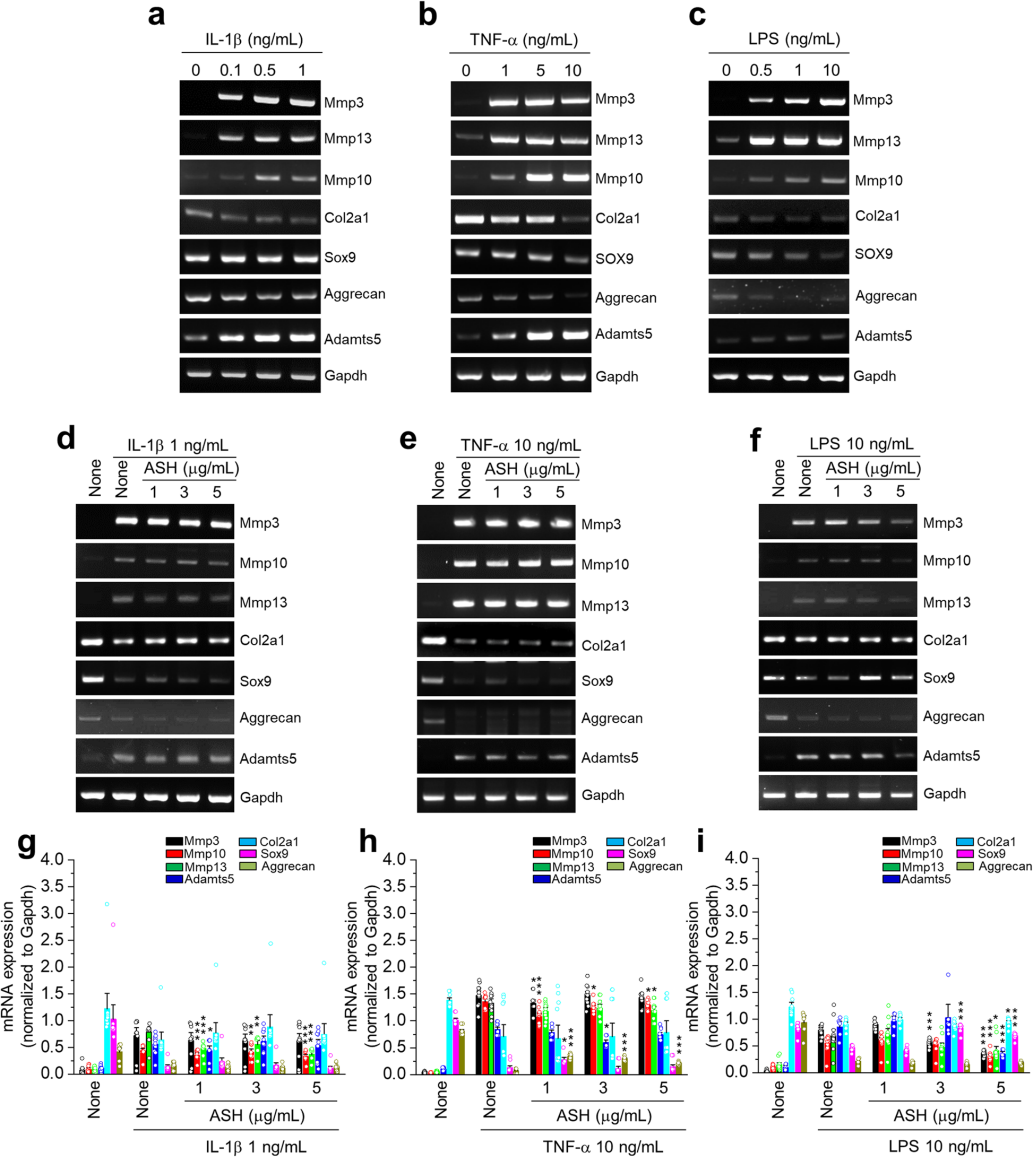
The effects of *A. sessiliflorus* Harms extract on pro-inflammatory cytokines and inflammatory mediator-induced anabolic or catabolic expression in primary culture articular chondrocytes. Primary cultured articular chondrocytes were exposed to (**a**) IL-1β (0–1 ng/mL), (**b**) TNF-α (0-10 ng/mL), (**c**) and LPS (0–10 ng/mL) for 24 h. Thereafter, mRNA expression of anabolic or catabolic factors was analyzed. In addition, chondrocytes were exposed to ASH (0–5 µg/mL) in the absence or presence of (**d**) IL-1β (1 ng/mL), (**e**) TNF-α (10-ng/mL), and (**f**) LPS (10 ng/mL) for 24 h, mRNA expression of anabolic or catabolic factors was analyzed, and semi-quantification results are presented in (**g**, **h**) and (**i**). The results are representative of at least six independent experiments. Values are presented as mean ± standard error of the mean (SEM) (*P < 0.05, **P < 0.01, and ***P < 0.001 vs. IL-1β, TNF-α, or LPS only), as analyzed via one-way ANOVA

The effects of the ASH extract on pro-inflammatory cytokines and inflammatory mediator-induced anabolic or catabolic expression were studied (Fig. 5). We investigated the effects of IL-1β, TNF-α, and LPS on anabolic and catabolic expression in primary cultured mouse chondrocytes. The results revealed that the pro-inflammatory cytokines (IL-1β, TNF-α) and inflammatory mediators (LPS) enhanced the catabolic factors (*Mmp3*, *Mmp13*, *Mmp10*, *Adamts5*) while decreasing anabolic factors (*Col2a1*, *SOX9*, *Aggrecan*) (Fig. 5a, b, c). When co-treated with ASH and IL-1β, the reduction in *Col2a1*, *Sox9*, and *Aggrecan* expression was marginally recovered (Fig. 5d, g). In addition, ASH reduced TNF-α-mediated *Mmp10* overexpression significantly (Fig. 5e, h). Notably, ASH attenuated the LPS-mediated *Mmp3*, *Mmp10*, *Mmp13*, and* Adamts5* expression significantly (Fig. 5f, i). In addition, ASH restored the LPS-mediated downregulation of *Sox9* expression (Fig. 5f, i).

### The effects of *A. sessiliflorus* Harms extract on NO production and NF-κB activation

**Fig. 6:**
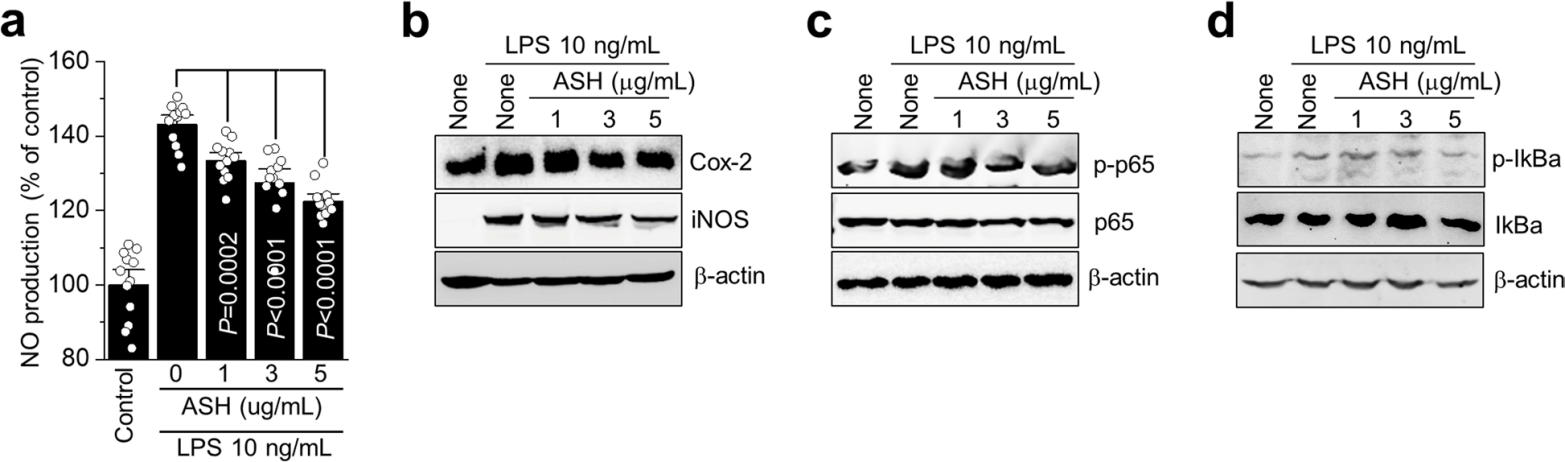
The inhibitory effects of *A. sessiliflorus* Harms extract on NO production and NF-kB activation in LPS-induced primary culture articular chondrocytes. Primary cultured articular chondrocytes were exposed to various concentration of ASH in the presence or absence of LPS. (**a**) NO production was measured, and protein levels of (**b**) Cox-2 and iNOS, (**c**) p-p65 and p65, and (**d**) p-IkBa and IkB were analyzed using Western blotting

The inhibition of NO production and the expression of proteins associated with ASH were studied in relation to NF-κB activity (Fig. 6). Compared to the LPS only group, NO production significantly decreased in proportion to the concentration of ASH extract (Fig. 6a). The expression of NF-κB activity-related components following ASH treatment was validated via Western blotting. As the concentration of ASH increased, the production of inflammation-inducing enzymes iNOS and COX-2 decreased (Fig. 6b). In general, IkBa is bound to NF-κB, but it does not act as a transcription factor. However, IkBa is phosphorylated by stimuli such as LPS to promote the synthesis of various inflammatory mediators. Here, phosphorylation of IkBa was inhibited using ASH treatment, and phosphorylation of NF-κB p65 was also inhibited (Fig. 6c and d). These results confirmed that the ASH extract suppresses NO production and inflammation by reducing the NF-κB activation response in an inflammatory environment.

### Effects of pimaric and kaurenoic acids in pro-inflammatory cytokine-induced catabolic expression

**Fig. 7:**
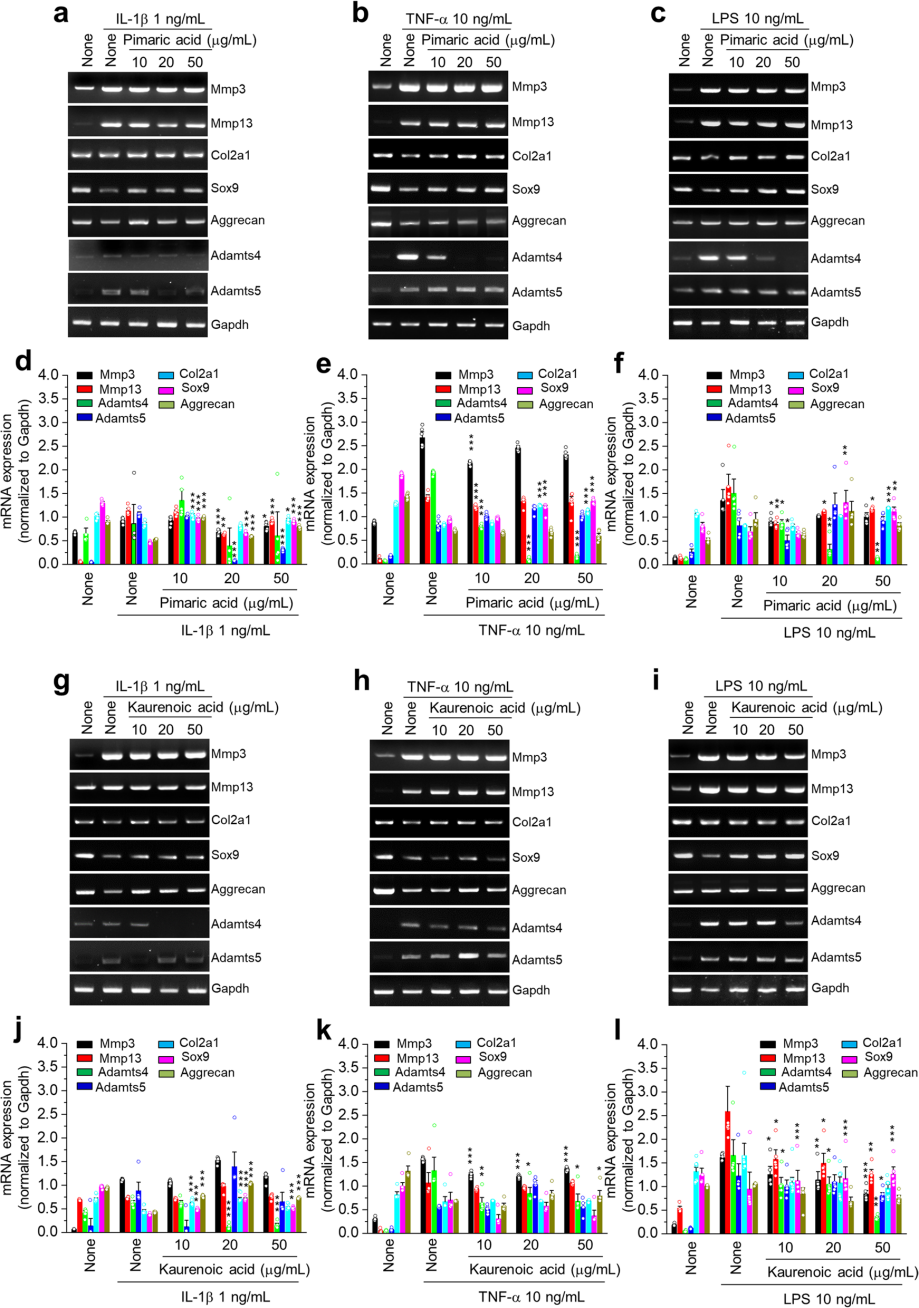
The inhibitory effects of pimaric and kaurenoic acids in the pro-inflammatory cytokine-induced catabolic expression in primary culture articular chondrocytes. Primary cultured articular chondrocytes were exposed to pimaric acid (0–50 µg/mL) in the absence or presence of (**a**) IL-1β (1 ng/mL), (**b**) TNF-α (10 ng/mL) and (**c**) LPS (10 ng/mL) for 24 h. Thereafter, mRNA expression of anabolic or catabolic factors was analyzed. Chondrocytes were exposed to kaurenoic acid (0–50 µg/mL) in the absence or presence of (**g**) IL-1β (1 ng/mL), (**h**) TNF-α (10 ng/mL), and (**i**) LPS (10 ng/mL) for 24 h. Thereafter, mRNA expression of anabolic or catabolic factors was analyzed. In addition, semi-quantification results are presented in (**d**, **e**, **f**, **j**, **k**, and **l**). The results are representative of at least six independent experiments. Values are presented as mean ± standard error of the mean (SEM) (*P < 0.05, **P < 0.01, and ***P < 0.001 vs. IL-1β, TNF-α, or LPS only), as analyzed via one-way ANOVA

The anti-inflammatory effects of pimaric and kaurenoic acids, the two major phytochemicals in ASH (Fig. 1 and Table 1), were examined in inflammatory cells generated by pro-inflammatory cytokines (IL-1β, TNF-α, and LPS) (Fig. 7). The expression of cartilage matrix synthesis (*Col2a1*, *Sox9*, *Aggrecan*) and degradation factors (*MMP-3*, *MMP-13*, *Adamts4*, *Adamts5*) corroborated the recovery or reduction in trend, with the majority of the degradation factors in pimaric acid (Fig. 7a, b, c, d, e, f) and kaurenoic acid (Fig. 7g, h, i, j, k, l) treatment groups. These results suggested that pimaric and kaurenoic acids exert protective effects on chondrocytes by decreasing cartilage matrix breakdown factors.

### The effects of pimaric and kaurenoic acids on the Th17-polarization of CD4^+^ T cells

**Fig. 8:**
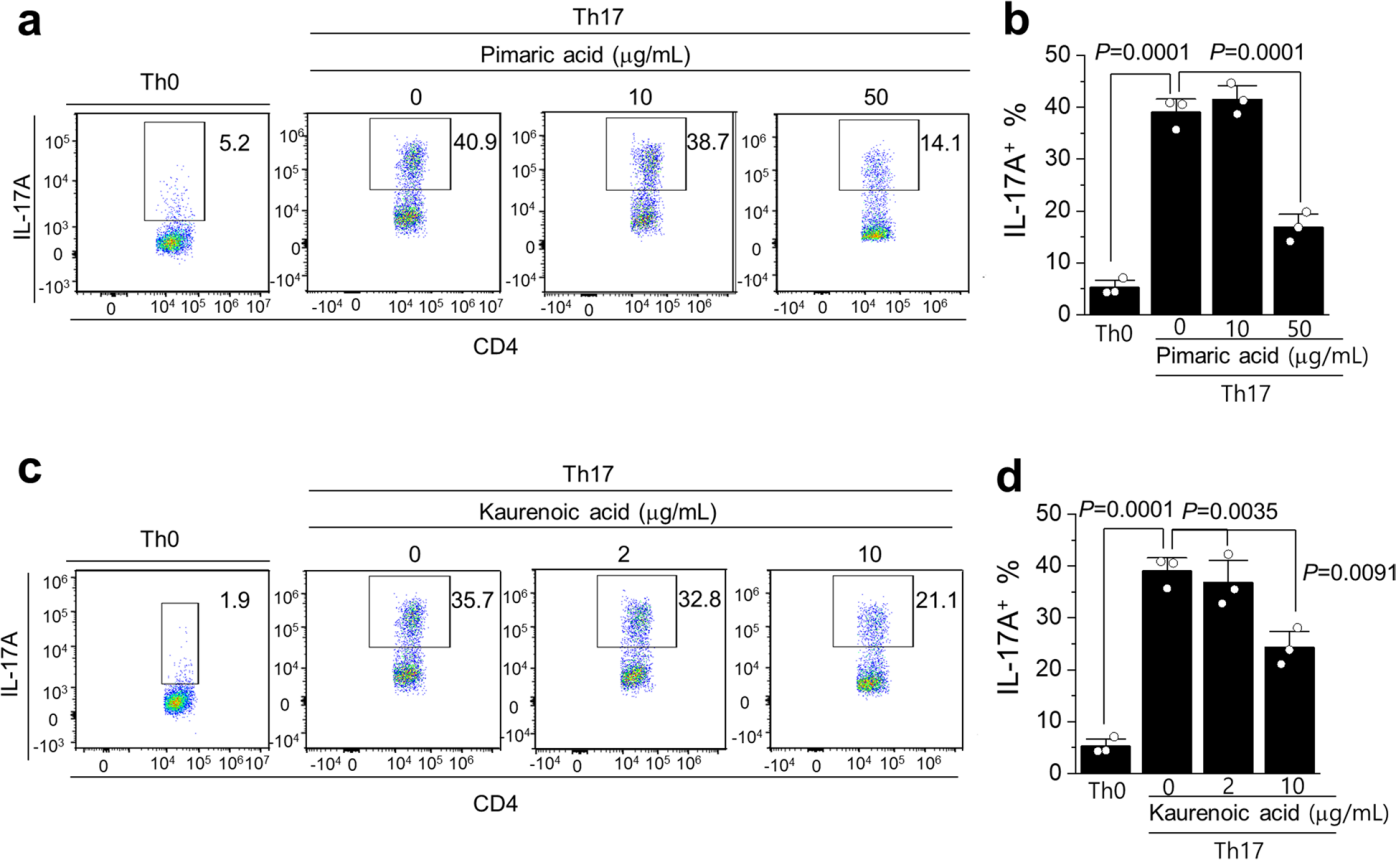
Pimaric and kaurenoic acids suppress Th17 polarization of CD4^+^cells. Splenocytes from C57BL/6 mice were stimulated with 1 μg/mL anti-CD3/CD28 in the presence or absence of IL-6 (50 ng/mL) and TGF-β (1 ng/mL) for 72 h. Either (**a** and **b)** pimaric or (**c** and **d)** kaurenoic acid was added at indicated concentrations. The expression of IL-17A among CD4^+^ T cells was analyzed using FACS. Representative plots and graphs are shown with the mean ± SEM. Data are representative of three independent experiments

Considering the clinical relevance of IL-17A in patients with RA [42], we further examined whether pimaric and kaurenoic acids affected IL-17A-producing CD4^+^ T cells (Th17). Splenocytes from C57BL/6 mice were stimulated with anti-CD3/CD28 and Th17-polarizing cytokines (IL-6/TGF-β) in the presence or absence of these chemical components. We observed that cytokine treatment markedly increased the frequency of IL-17A^+^ cells among CD4^+^ T cells compared to that of unpolarized splenocytes (Th0) (Fig. 8). Upon pimaric acid treatment at 50 μg/mL, there were 2.5-fold fewer IL-17A^+^ cells than in untreated splenocytes (Fig. 8a, b). Similarly, the expression of IL-17A in CD4^+^ T cells was markedly lower in kaurenoic acid-treated splenocytes than in untreated splenocytes (Fig. 8 c, d). None of the chemicals exerted significant effects at lower concentrations (Fig. 8).

### Annotation of pathway and construction of algorithm-based gene regulatory networks

**Fig. 9:**
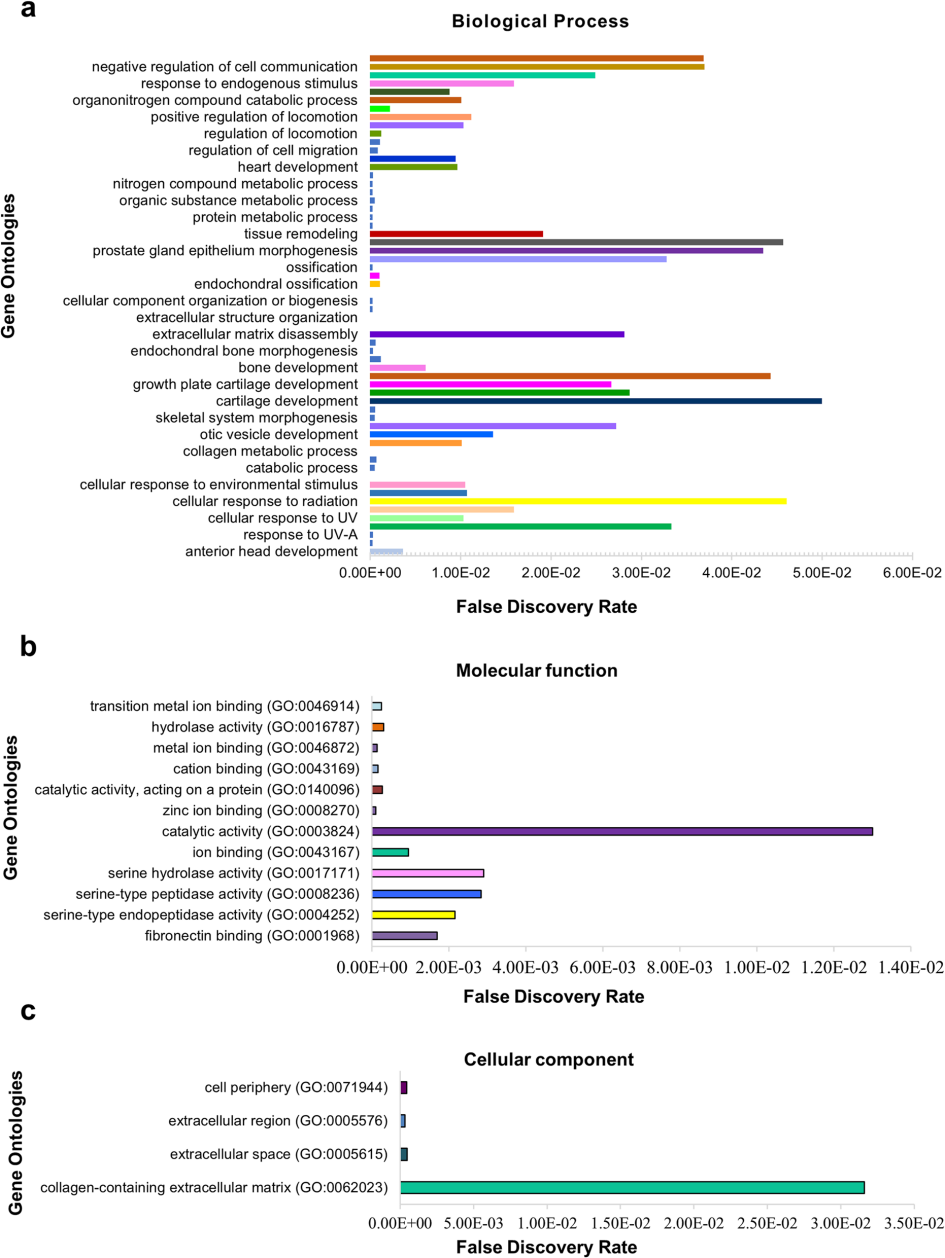
Ontology and functional association annotation of putative genes (Mus musculus) using Fisher’s exact test. The GO enrichment analysis of (**a**) biological processes, (**b**) molecular functions, and (**c**) cellular components consisting of 76 functional groups

We used two complementary models of gene function: Gene Ontology (GO) and pathway ontology. The biological process (60), molecular function (12), and cellular components (4) of the functional groups were included in the GO enrichment analysis (Fig. 9). We identified the majority of selected genes potentially related to biological processes, such as cartilage and bone development, tissue remodeling, negative regulation of cell communication, and response to endogenous stimuli, while the ontologies related to molecular functions were catalytic activity and collagen-containing extracellular matrix, and the cellular component ontology was identified as collagen-containing extracellular matrix. Pathway analysis revealed that 14 of the 16 stated genes in the sample had at least one striking 208 pathways. Based on the *p*-values, 25 relevant pathways were identified (Table 3). The following pathways were selected for further analysis: collagen degradation, extracellular matrix degradation, activation of matrix metalloproteinases, extracellular matrix organization, interleukin-4 and interleukin-13 signaling, collagen formation, and zinc influx into cells via the SLC39 gene family.

**Table 3:** The 25 most relevant pathways sorted by *p*-value

Pathway name	Entities				Reactions	
	**Found**	**Ratio**	***p*-value**	**FDR**	**Found**	**Ratio**
Collagen degradation	10/69	0.005	2.11e-15	7.55e-14	24/34	0.002
Degradation of the extracellular matrix	12/148	0.01	2.22e-15	7.55e-14	77/105	0.007
Activation of matrix Metalloproteinases	8/35	0.002	4.63e-14	1.02e-12	22/27	0.002
Extracellular matrix organization	12/328	0.022	2.37e-11	4.02e-10	115/319	0.022
Interleukin-4 and interleukin-13 signaling	6/211	0.014	1.59e-05	2.07e-04	1/47	0.003
Assembly of collagen fibrils and other multimeric structures	4/67	0.004	1.59e-05	2.07e-05	7/26	0.002
Collagen formation	4/104	0.007	1.50e-04	0.001	29/77	0.005
RUNX2 regulates genes involved in cell migration	2/14	9.23e-04	6.17e-04	0.005	2/7	4.93e-04
Transcriptional regulation of testis differentiation	2/21	0.001	0.001	0.01	14/18	0.001
Extranuclear estrogen signaling	3/11	0.007	0.003	0.018	1/39	0.003
Defective B3GALTL causes PpS	2/39	0.003	0.005	0.025	1/1	7.41e-05
O-glycosylation of TSR domain containing proteins	2/41	0.003	0.005	0.025	2/2	1.41e-04
Signaling by interleukins	6/658	0.043	0.006	0.026	1/505	0.036
Transcriptional regulation by RUNX2	3/147	0.01	0.006	0.026	3/84	0.006
EPH-ephrin-mediated repulsion of cells	2/55	0.004	0.009	0.036	1/9	6.34e-04
Diseases associated with O-glycosylation of proteins	2/78	0.005	0.017	0.069	1/9	6.34e-04
Zinc influx into cells by the SLC39 gene family	1/11	7.25e-04	0.028	0.084	1/6	4.23e-04
EPH-ephrin signaling	2/102	0.007	0.028	0.085	1/56	0.004
MAPK6/MAPK4 signaling	2/106	0.007	0.03	0.091	1/40	0.003
EGFR transactivation by gastrin	1/13	8.57e-04	0.033	0.092	2/6	4.23e-04
Zinc transporters	1/16	0.001	0.04	0.092	1/13	9.16e-04
O-linked glycosylation	2/133	0.009	0.046	0.092	2/27	0.002
Cytokine signaling in immune system	6/1036	0.068	0.047	0.095	1/740	0.052
Gastrin-CREB signaling pathway via PKC and MAPK	1/24	0.002	0.06	0.12	2/9	6.34e-04

**Fig. 10:**
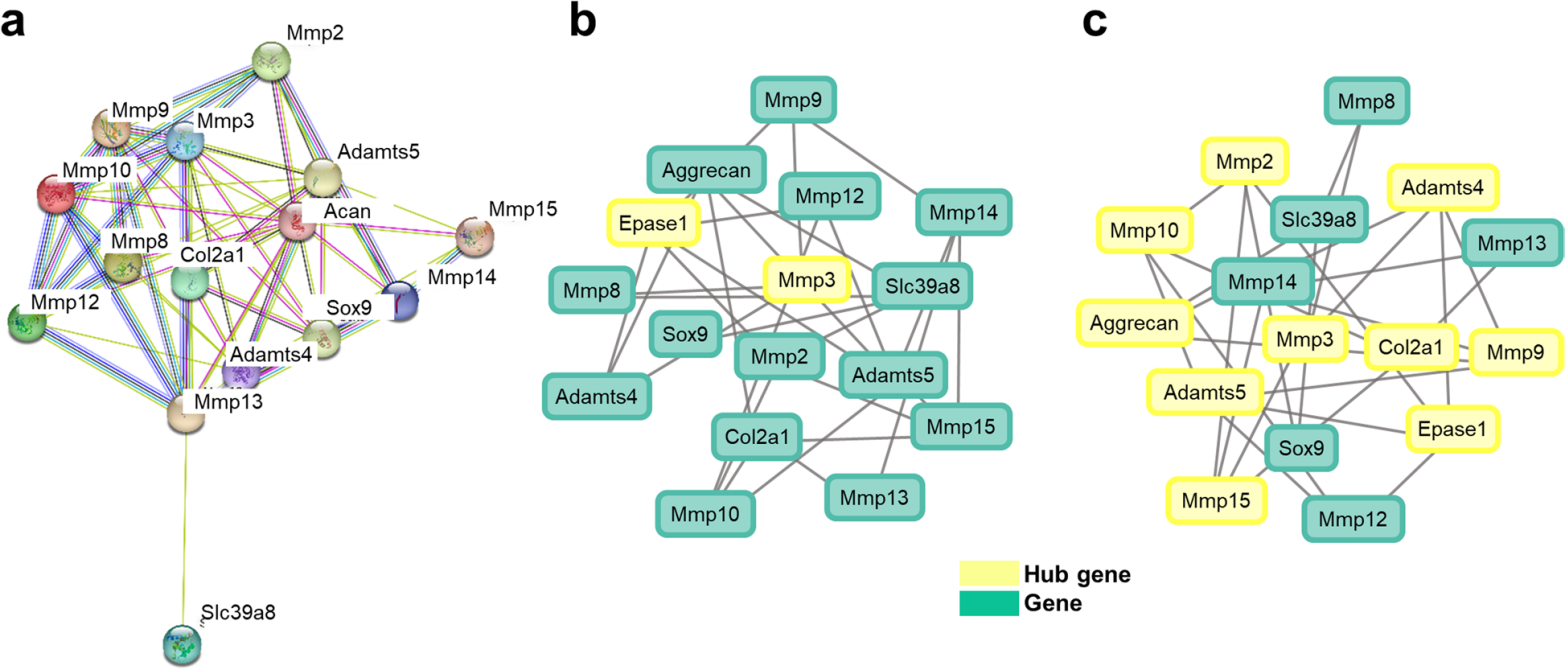
String network annotation and network inference analysis of selected genes. (**a**) An abstracted network composed of biological functions, molecular processes, cellular components, and pathways. The enriched functional GO and pathways with the cluster of genes, (**b**) the PCACMI method using the Gaussian kernel density estimator, and the (**c**) CMI2NI method using Kullback–Leibler for statistical analysis were incorporated to build gene networks and for the selection of hub genes

The protein–protein interaction via STRING network annotation revealed a total of 15 nodes with an average degree of 8.27 and a protein–protein interaction (PPI) enrichment *p*-value of < 1.0e-16. This suggests that the selected proteins have more interactions among themselves than would be expected for a random set of proteins of the same size and degree distribution drawn from the genome. This enrichment indicated that the proteins are at least partially biologically connected as a group (Fig. 10a). Furthermore, based on pertinent gene expression and optional hub gene information, we conducted statistical analysis to build gene networks. To reconstruct the gene regulatory networks based on conditional CMI, we first attempted to find the path consistency algorithm (PCA) to identify dependent pairings of variables. We found two hub genes using PCACMI: the first node from *Epase1* stringed with its neighbors *Adamts4*, *Adamts5*, *Mmp9*, *Mmp12*, and *Mmp2*. In contrast, the second node from *Mmp3* stringed with its neighbors *Adamts4*, *Aggrecan*, *Mmp9*, *Mmp15*, and *Mmp8* (Fig. 10b). The CMI2NI approach improves the PCACMI method by considering the Kullback–Leibler divergence from the joint probability density function of the target variables to the interventional PDFs, reducing the dependence between the two variables of interest. Hub genes with the highest linkages were identified in each module. Ten hub genes were identified using CMI2NI (Fig. 10c). To efficiently calculate the CMI2 estimates, PCA as used in PCACMI was also employed in CMI2NI. Two variables were considered independent in the PCA phases of CMI2NI if the associated CMI2 estimate was less than the set threshold α (0.04).

## Discussion

RA is associated with an increased risk of various health disorders including inflammation, which has additional social and economic implications. Patients with RA have mortality rates more than twice as high as those in the general population, and this difference appears to be increasing. Similar to other autoimmune illnesses, both early and later stages of RA involve abnormal T-cell activation. CD4^+^ T effector cells (Th-1, 2, and 17) observed in RA synovial joints are closely related to RA pathogenesis [43–45]. There is currently no cure for RA; however, the primary goal of any treatment strategy is to identify the disease earlier and promptly achieve a low disease activity state (LDAS). Prescription drugs are useful in reducing stiffness and pain; however, they do not slow the progression of the illness [46]. Skin rashes, urticaria, headaches, dizziness, sleepiness, hypertension, and edema can all be induced by arthritis treatment. Therefore, it is essential to identify effective alternative treatments with fewer side effects [27]. Recent ethnopharmacological research on RA has focused on the therapeutic targets and anti-RA benefits of various THM dosage forms [12, 27, 47]. The present study provides comprehensive information on the systematic functions of pimaric and kaurenoic acids from *A. sessiliflorus* Harms for the treatment of RA, which has not been previously reported.

In RA, fibrin networks may function as a matrix for inflammatory cells during pannus development [48]. The two primary mechanisms that cause cartilage deterioration in RA are invasion by the pannus tissue (invasive vascularized connective tissue) and the catabolic effects of inflammatory cytokines and proteases [49, 50]. Mast cells may also have both pro-inflammatory and anti-inflammatory functions in RA. Notably, the total number of mast cells and degranulated mast cells increased in the untreated CIA group; however, they decreased significantly in the treated group (*p* = 0.0434). CIA has become a frequently employed and valuable murine animal model for studying the etiology of the pathogenic mechanisms of RA and assessing prospective treatment therapies [51, 52]. The CIA model simulating RA in the knee and ankle significantly triggered the invasion of immune cells such as eosinophils, neutrophils, macrophages, and T cells into joint tissues, the secretion of cytokines and MMPs, and a change in T-cell subsets [52–54]. Compared to the untreated groups, eosinophil levels were considerably lower in the treated groups. Immunostaining for CDr15 also revealed alterations in the neutrophil counts in the experimental groups, whereas those in the knees of the treated group substantially decreased. A rapid increase in neutrophil infiltration accelerates LPS. LPS binding to toll-like receptor 4 (TLR4) initiates the NF-κB signaling pathway, resulting in the production of inflammatory factors IL-6, IL-1β, and TNF-α [55], which play critical roles in the pathogenesis of RA. Targeted therapies for these cytokines show dramatic therapeutic effects on RA [56]. COX-2, iNOS, and their pro-inflammatory mediators play important roles in promoting the inflammatory response of NF-κB [57–59]. ASH inhibits the production of NO and prostaglandin E2 (PGE2) by suppressing the expression of iNOS and COX-2 in the LPS group. Deregulation of NF-κB and IκB phosphorylation promotes the initiation of various inflammatory diseases. We found that ASH inhibited IkappaB kinase (IKK)-mediated phosphorylation of NF-κB and p65. NF-κB is primarily activated via IKK-mediated phosphorylation of inhibitory molecules such as IkB-α. Phosphorylation of NF-κB proteins, such as p65, inside their transactivation domain by a variety of kinases in response to different stimuli is also required for optimal induction of NF-κB target genes [60].

We selected seven major pathways and the functional associations of gene ontologies to unravel their complexities and in-depth mechanisms. Annotations were developed using a wide range of evidential sources (PCACMI and CMI2NI), which may be used to determine the relative reliability of various annotations. Arthritis is unavoidably associated with proteoglycan degradation; however, the disintegration of the collagen network is irreversible and contributes to joint function loss. Proteoglycan aggregates are composed of non-covalent interactions between aggrecan, hyaluronate, and link proteins, which constitute the main components of cartilage: a type II collagen fiber network with small, associated proteoglycans [61]. The diameter and spatial arrangement of collagen fibrils are determined by the species, tissue type, and developmental stage. It has been proposed that the collagen fibril proportion is such that it maintains a fine balance between protection and susceptibility. In this study, we found that *ADAMTS4* and *ADAMTS5* were the main proteases responsible for the degradation of proteoglycans. Proteolysis by MMPs damages cartilage whose expression influences numerous endogenous mediators including cytokines, growth factors, prostaglandins, oxygen species, and neuropeptides [62]. Continuous degradation leads to the formation of gelatin. The degradation of collagen types other than I–III is not well characterized; however, it is believed to follow a similar mechanism. Collagenases I, II, and III can initiate the intrahelical cleavage of the major fibril-forming collagens I, II, and III at neutral pH and are thus thought to define the rate-limiting step in normal tissue remodeling events. Collagenases cleave collagen alpha chains at a single conserved Gly-Ile/Leu site, which is approximately three-quarters of the length of the molecule from the N-terminus [63]. Collagen must unfold locally into non-triple helical areas for possible collagenolysis. Circular dichroism and differential scanning calorimetry observations show substantial variability along the collagen fibers [64]. The cleavage site is characterized by the motif G(I/L)(A/L); the GI/L bond is cleaved (in collagen type I: G953-I954; UniProt canonical alpha chain sequences); however, it is unclear why this position acts as the cleavage site, since the motif occurs at several other places in the chain.

MMPs, previously referred to as matrixins because of their role in the degradation of the extracellular matrix (ECM), are zinc- and calcium-dependent proteases belonging to the metzincin family. MMPs are controlled by transcription, cellular location (most are not active until secretion), activating proteinases of other MMPs, and metalloproteinase inhibitors such as tissue inhibitors of metalloproteinases. MMPs are well-recognized for their function in ECM breakdown and removal. Furthermore, ECM and other cell surface molecules can be cleaved, releasing ECM-bound growth factors and various non-ECM protein substrates [65]. Thus, MMPs are involved in several physiological and pathological processes such as arthritis and tissue remodeling [66].

*AGGRECAN* is the predominant ECM proteoglycan in cartilage that interacts with hyaluronan to form large aggregates, which are responsible for the ability of tissues to resist compressive loads. This function is related to the structure of aggrecan and, in particular, to the large number of chondroitin sulfate chains present in its core. Polymorphisms in the human *AGGRECAN* gene region, which encodes the CS1 domain, can cause variations in the amount of chondroitin sulfate substitution of aggrecan in the population. This suggests that the functional characteristics of *AGGRECAN* may differ between individuals, and those with an inferior aggrecan structure may be more susceptible to prompt intervertebral disc or articular cartilage deterioration [67]. The results showed that pimaric and kaurenoic acids effectively inhibited the expression of cartilage matrix synthesis factors (*Col2a1*, *Aggrecan*) and degradation factors (*Mmp3*, *Mmp13*, *Sox9*, *Adamts4*, *Adamts5*). Previous in vivo studies have demonstrated that MMPs are involved in the degradation of several matrix components, and that the activities of MMPs are regulated by hormones and cytokines [66, 68].

## Conclusions

**Fig. 11:**
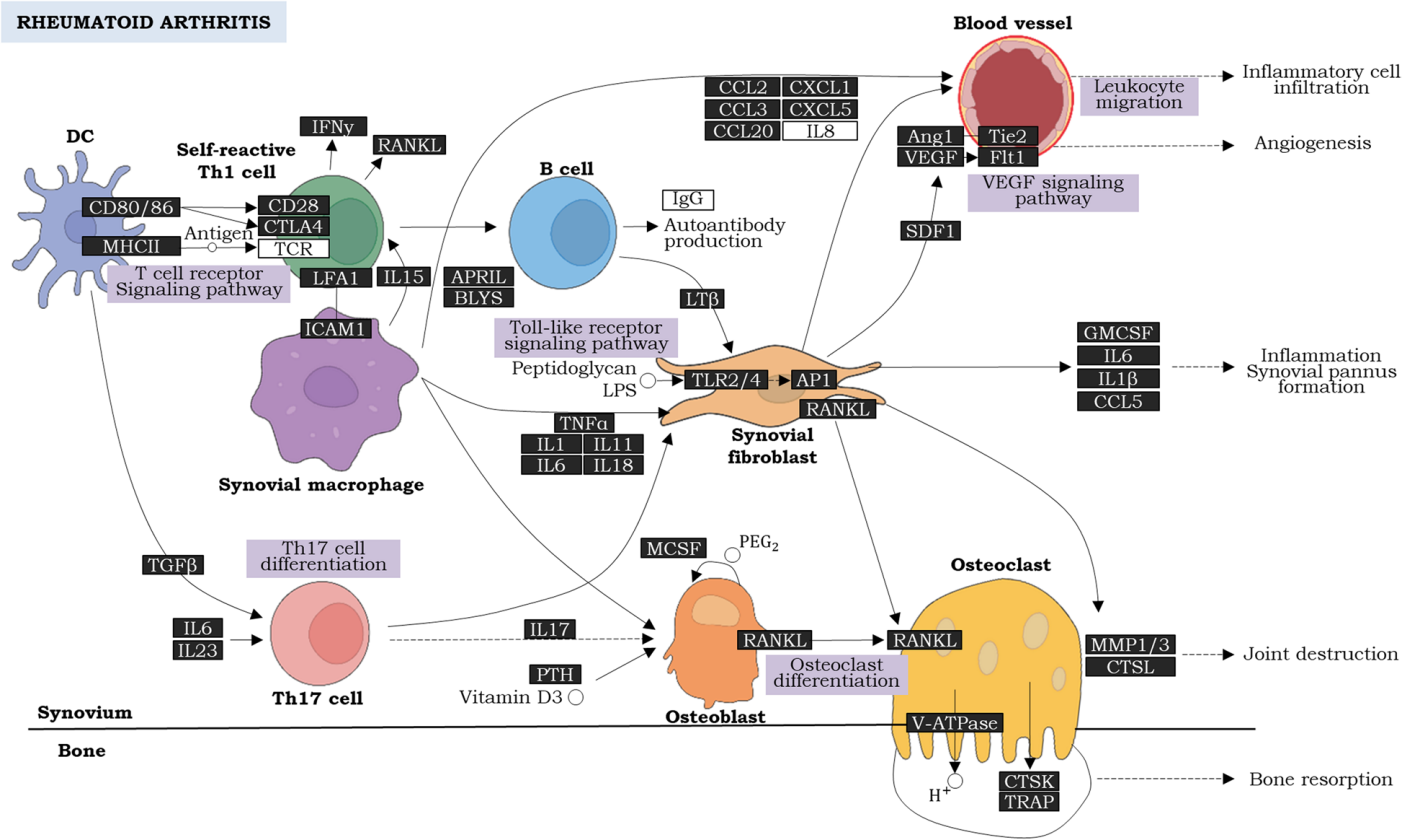
The systematic pathway illustration of rheumatoid arthritis: chronic inflammation disrupts bone remodeling, resulting in gradual bone loss. The aberrant activation of the immune system raises the levels of pro-inflammatory cytokines and chemokines, which can increase synovial angiogenesis and leukocyte infiltration. The synovium generates a hyperplastic pannus with infiltrating macrophage- and fibroblast-like synoviocytes and invades joints by secreting proteinases and stimulating osteoclast differentiation (modified, KEGG Copyright Permission from Kanehisa Laboratories; no.: 230744)

Research until date has focused exclusively on the extracted components of ASH; however, the animal models used to study these effects were not post-traumatic CIA models, which are more well-suited for the study of RA. Furthermore, information on the active components in ASH was unavailable. In this study, pimaric and kaurenoic acids, extracted from ASH using supercritical CO_2_, were demonstrated to be the two major components involved in the pathogenesis of RA. Compared to the arthritis group, cartilage erosion, proteoglycan loss, synovitis, and subchondral plate thickness were reduced in the ASH treatment group. These phenomena are directly linked to specific inhibitory actions against IL-6-mediated anabolic and catabolic imbalances (Fig. 11). These results indicate that ASH has vast potential for the treatment of RA. Nevertheless, further investigations are important to unravel the impact of individual active substances on RA.

### Supplementary Information

**Additional file 1: Supplementary Figure 1.** Effects of ASH on cell viability in primary articular chondrocyte cultures. (a) Primary cultured chondrocytes were exposed to ASH (0 – 20 μg/mL) for 24 h. Also, ASH (0 – 5 μg/mL) was exposed to (b) IL-1β (1 ng/ mL), (c) TNF-α (10 ng/ mL), and (d) LPS (10 ng/ mL) for 24 h. Further, MTT assay was performed. **Supplementary Figure 2.** Effects of ASH on anabolic and catabolic factor expression in primary cultures of articular chondrocytes. Primary chondrocytes were exposed to ASH (0 – 5 μg/mL) for 24 h, further, the mRNA expression of (a) cartilage degradation factors, (b) Mmps, and (c) anabolic/catabolic factors were analyzed. **Supplementary Figure 3.** Inhibitory effects of ASH on ankle immune cell infiltration in a CIA (rheumatoid arthritis) model. The total mast cell and degranulated mast cells were analyzed using experimental mice cartilage. (a) Mast cell number and activity in the knee were analyzed via toluidine blue staining, (b) total or degranulated mast cells were counted. **Supplementary Figure 4.** Effects of pimaric acid and kaurenoic acid on the viability of primary articular chondrocytes. Primary cultured chondrocytes were exposed to (a) pimaric acid (0 – 100 μg/mL) and (b) kaurenoic acid (0 – 100 μg/mL) for 24 h. Further, MTT assay was performed. ** <0.01 and ***<0.001. **Supplementary Figure 5.** Effects of pimaric acid and kaurenoic acid on the expression of anabolic and catabolic factors in primary articular chondrocytes. Primary chondrocytes were exposed to (a) pimaric acid (0 – 50 μg/mL) and (b) kaurenoic acid (0 – 50 μg/mL) for 24 h. Further, the mRNA expression of Mmps and anabolic/catabolic factors were analyzed. **Supplementary Figure 6.** Uncropped images of the original conventional RT-PCR data are in Figures 5a, b, c. **Supplementary Figure 7.** Uncropped images of the original conventional RT-PCR data are in Figures 5d, e, f. **Supplementary Figure 8.** Uncropped images of the original western blot in Figure 6b, c, and d. **Supplementary Figure 9.** Uncropped images of the original conventional RT-PCR data are in Figures 7a, b, c. **Supplementary Figure 10.** Uncropped images of the original conventional RT-PCR data are in Figures 7g, h, i.

## Data Availability

The datasets used and/or analyzed during the current study are available from the corresponding author upon reasonable request.

## Abbreviations

ASH: Acanthopanax sessiliflorus Harms
RA: Rheumatoid arthritis
CIA: Collagen-induced arthritis
NSAIDs: Nonsteroidal anti-inflammatory drugs
MMP: Matrix metalloproteinase
GC-MS: Gas chromatography-mass spectrometry
PEG-400: Polyethylene glycol 400
qRT-PCR: Quantitative real-time polymerase chain reaction
OARSI: Osteoarthritis Research Society International
EASE: Expression Analysis Systematic Explorer
GO: Gene Ontology
PCACMI: Path consistency algorithm based on conditional mutual information
CMI2NI: Conditional mutual inclusive information-based network inference
LDAS: Low disease activity state
THMs: Traditional herbal medicines
PGE2: Prostaglandin E2
ECM: Extracellular matrix
NI: Non-immunized
TLR4: Toll-like receptor
NF-κB: Nuclear factor-κB
EDTA: Ethylenediaminetetraacetic acid
Col1a1: Collagen type 1 alpha 1
Il-6: Interleukin-6
Il-17: Interleukin-17
