# Supplementary material for: Inhibitory effects of Acanthopanax sessiliflorus Harms extract on the etiology of rheumatoid arthritis in a collagen-induced arthritis mouse model
Source: Arthritis Res Ther. 2024 Jan 2;26:11. doi: 10.1186/s13075-023-03241-1 (PMC10763440; doi:10.1186/s13075-023-03241-1)
Supplement: Supplementary file 1 — Additional file 1: Supplementary Figure 1. Effects of ASH on cell viability in primary articular chondrocyte cultures. (a) Primary cultured chondrocytes were exposed to ASH (0 – 20 μg/mL) for 24 h. Also, ASH (0 – 5 μg/mL) was exposed to (b) IL-1β (1 ng/ mL), (c) TNF-α (10 ng/ mL), and (d) LPS (10 ng/ mL) for 24 h. Further, MTT assay was performed. Supplementary Figure 2. Effects of ASH on anabolic and catabolic factor expression in primary cultures of articular chondrocytes. Primary chondrocytes were exposed to ASH (0 – 5 μg/mL) for 24 h, further, the mRNA expression of (a) cartilage degradation factors, (b) Mmps, and (c) anabolic/catabolic factors were analyzed. Supplementary Figure 3. Inhibitory effects of ASH on ankle immune cell infiltration in a CIA (rheumatoid arthritis) model. The total mast cell and degranulated mast cells were analyzed using experimental mice cartilage. (a) Mast cell number and activity in the knee were analyzed via toluidine blue staining, (b) total or degranulated mast cells were counted. Supplementary Figure 4. Effects of pimaric acid and kaurenoic acid on the viability of primary articular chondrocytes. Primary cultured chondrocytes were exposed to (a) pimaric acid (0 – 100 μg/mL) and (b) kaurenoic acid (0 – 100 μg/mL) for 24 h. Further, MTT assay was performed. ** <0.01 and ***<0.001. Supplementary Figure 5. Effects of pimaric acid and kaurenoic acid on the expression of anabolic and catabolic factors in primary articular chondrocytes. Primary chondrocytes were exposed to (a) pimaric acid (0 – 50 μg/mL) and (b) kaurenoic acid (0 – 50 μg/mL) for 24 h. Further, the mRNA expression of Mmps and anabolic/catabolic factors were analyzed. Supplementary Figure 6. Uncropped images of the original conventional RT-PCR data are in Figures 5a, b, c. Supplementary Figure 7. Uncropped images of the original conventional RT-PCR data are in Figures 5d, e, f. Supplementary Figure 8. Uncropped images of the original western blot in Figure 6b, c, and d. S [file 13075_2023_3241_MOESM1_ESM.docx]

**Supporting information**

**Inhibitory Effects of *Acanthopanax Sessiliflorus* Harms Extract on Rheumatoid Arthritis Etiology in a Collagen-Induced Arthritis Mouse Model**

Dahye Kim ^a,#^, Yunji Heo^b,#^, Mangeun Kim^b^, Godagama Gamaarachchige Dinesh Suminda^c^, Umar Manzoor^c^, Yunhui Min^c^, Minhye Kim^b^, Jiwon Yang^c^, Young-Jun Park^c,d^, Yaping Zhao^e^, Mrinmoy Ghosh^a,f*^, and Young-Ok Son^b,c*^


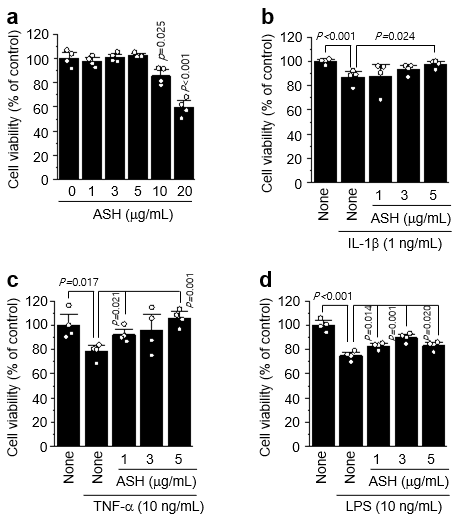


**Supplementary Figure 1**. Effects of ASH on cell viability in primary articular chondrocyte cultures. (a) Primary cultured chondrocytes were exposed to ASH (0 – 20 μg/mL) for 24 h. Also, ASH (0 – 5 μg/mL) was exposed to (b) IL-1β (1 ng/ mL), (c) TNF-α (10 ng/ mL), and (d) LPS (10 ng/ mL) for 24 h. Further, MTT assay was performed


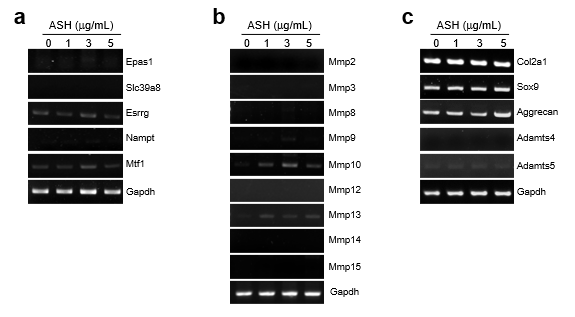


**Supplementary Figure 2.** Effects of ASH on anabolic and catabolic factor expression in primary cultures of articular chondrocytes. Primary chondrocytes were exposed to ASH (0 – 5 μg/mL) for 24 h, further, the mRNA expression of (a) cartilage degradation factors, (b) Mmps, and (c) anabolic/catabolic factors were analyzed


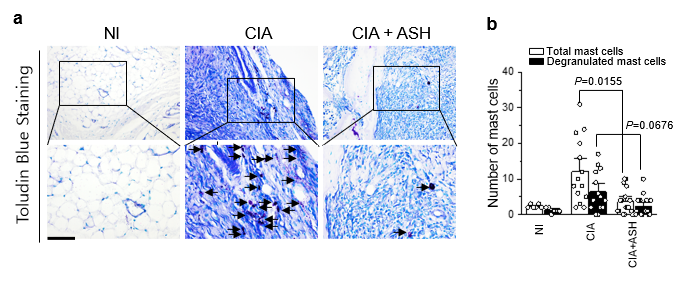


**Supplementary Figure 3.** Inhibitory effects of ASH on ankle immune cell infiltration in a CIA (rheumatoid arthritis) model. The total mast cell and degranulated mast cells were analyzed using experimental mice cartilage. (a) Mast cell number and activity in the knee were analyzed via toluidine blue staining, (b) total or degranulated mast cells were counted.


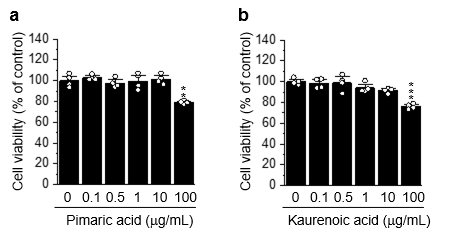


**Supplementary Figure 4.** Effects of pimaric acid and kaurenoic acid on the viability of primary articular chondrocytes. Primary cultured chondrocytes were exposed to (a) pimaric acid (0 – 100 μg/mL) and (b) kaurenoic acid (0 – 100 μg/mL) for 24 h. Further, MTT assay was performed. ** <0.01 and ***<0.001


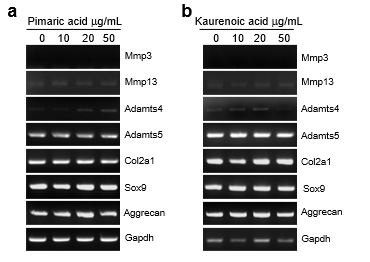


**Supplementary Figure 5.** Effects of pimaric acid and kaurenoic acid on the expression of anabolic and catabolic factors in primary articular chondrocytes. Primary chondrocytes were exposed to (a) pimaric acid (0 – 50 μg/mL) and (b) kaurenoic acid (0 – 50 μg/mL) for 24 h. Further, the mRNA expression of Mmps and anabolic/catabolic factors were analyzed


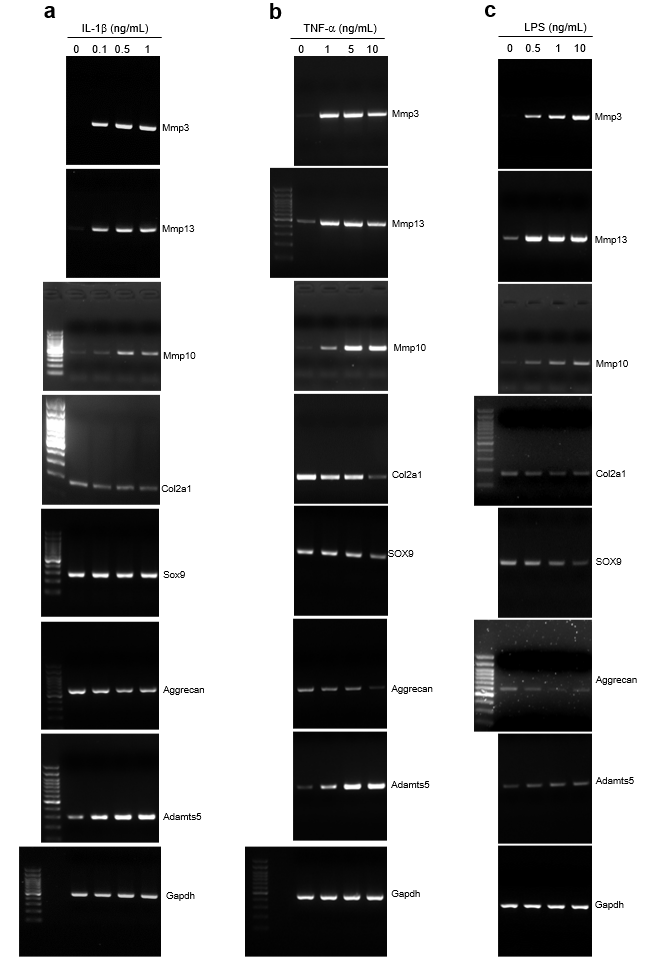


**Supplementary Figure 6.** Uncropped images of the original conventional RT-PCR data are in Figures 5a, b, c.


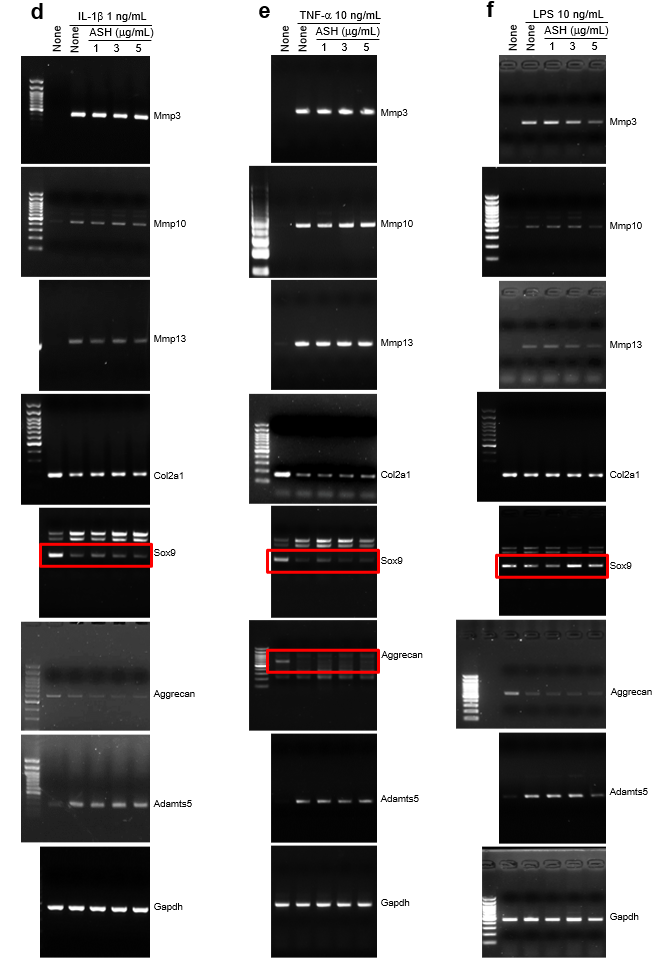


**Supplementary Figure 7.** Uncropped images of the original conventional RT-PCR data are in Figures 5d, e, f.


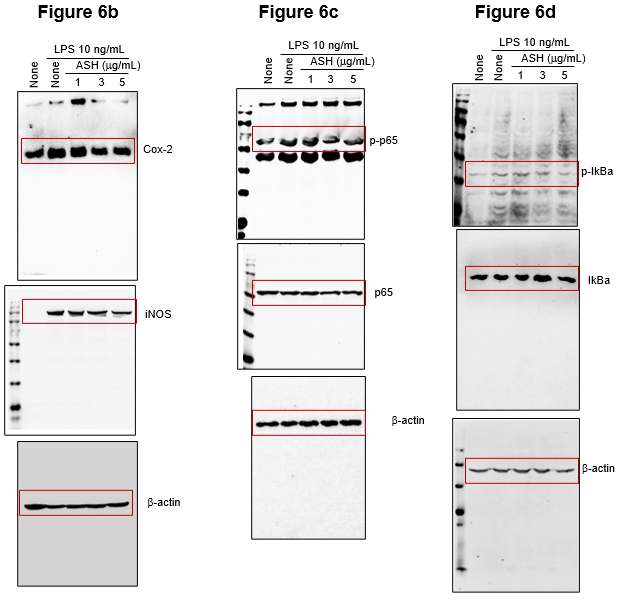


**Supplementary Figure 8.** Uncropped images of the original western blot in Figure 6b, c, and d.

**
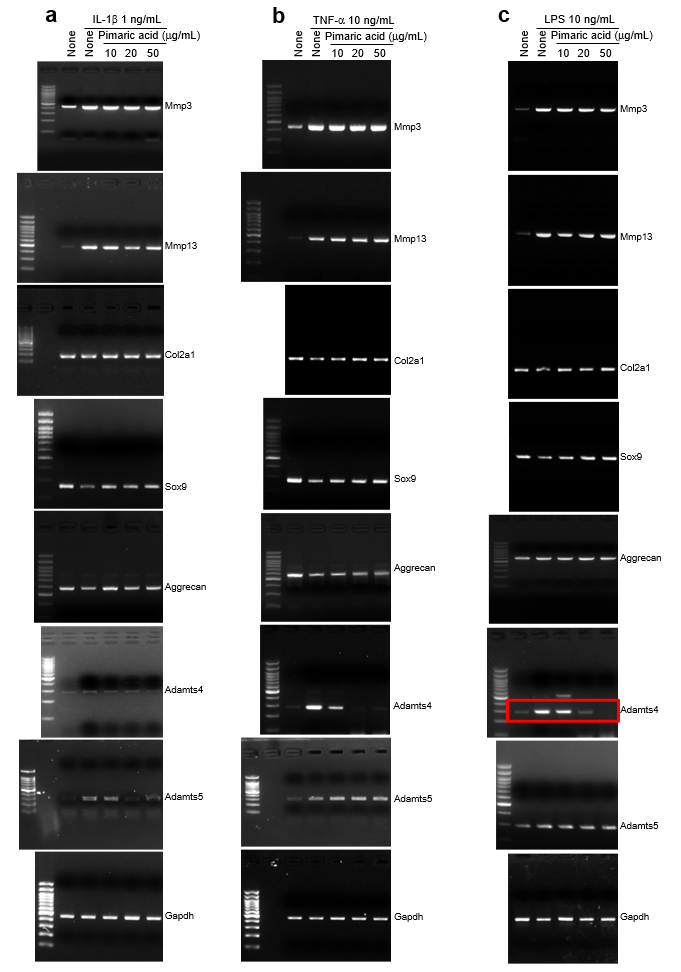
**

**Supplementary Figure 9.** Uncropped images of the original conventional RT-PCR data are in Figures 7a, b, c.

**
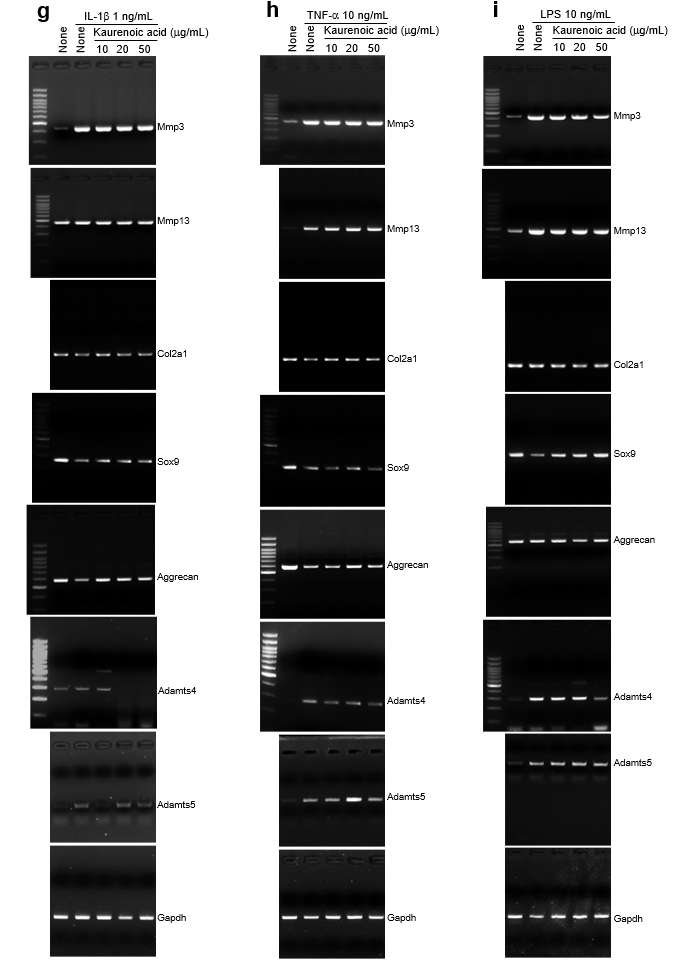
**

**Supplementary Figure 10.** Uncropped images of the original conventional RT-PCR data are in Figures 7g, h, i.
